# Sirtuin 1 genetic variation, energy balance and colorectal cancer risk by sex and subsite in the Netherlands Cohort Study

**DOI:** 10.1038/s41598-018-34728-6

**Published:** 2018-11-08

**Authors:** C. C. J. M. Simons, L. J. Schouten, R. W. Godschalk, F. J. van Schooten, P. A. van den Brandt, M. P. Weijenberg

**Affiliations:** 1 Department of Epidemiology, GROW – School for Oncology and Developmental Biology, Maastricht University, Maastricht, The Netherlands; 2 Department of Toxicology & Pharmacology, NUTRIM School of Nutrition and Translational Research in Metabolism, Maastricht University, Maastricht, The Netherlands; 3 Department of Epidemiology, CAPHRI – School for Public Health and Primary Care, Maastricht University Medical Center+, Maastricht, The Netherlands

## Abstract

Sirtuin 1 (SIRT1) is an energy-sensing protein, which may affect tumorigenesis. We used *SIRT1* variants as time-independent indicators of SIRT1 involvement in carcinogenesis and we studied two tagging *SIRT1* variants in relation to colorectal cancer (CRC) risk. We also evaluated known energy balance-related CRC risk factors within *SIRT1* genotype strata. The Netherlands Cohort Study includes 120,852 individuals and has 20.3 years follow-up (case-cohort: n_subcohort_ = 5000; n_CRC cases_ = 4667). At baseline, participants self-reported weight, weight at age 20, height, trouser/skirt size reflecting waist circumference, physical activity, and early life energy restriction. *SIRT1* rs12778366 and rs10997870 were genotyped in toenail DNA available for ~75% of the cohort. Sex- and subsite-specific Cox hazard ratios (HRs) showed that the rs12778366 CC versus TT genotype decreased CRC and colon cancer risks in women (HR_CRC_ = 0.53, 95% confidence interval: 0.30–0.94) but not men. Multiplicative interactions were observed between *SIRT1* variants and energy balance-related factors in relation to CRC endpoints, but the direction of associations was not always conform expectation nor specific to one genotype stratum. In conclusion, these results support SIRT1 involvement in colon cancer development in women. No conclusions could be made regarding a modifying effect of *SIRT1* variants on associations between energy balance-related factors and CRC risk.

## Introduction

Colorectal cancer (CRC) is one of the most common cancers in men and women constituting close to 10% of the 14.1 million incident cancer cases worldwide in 2012^1^. It is important to increase our understanding of the biological mechanisms involved in CRC development and those underlying the association between energy balance-related modifiable factors and CRC risk^2^. Quantifying the effect of modifiable energy balance-related CRC risk factors against a background of relevant biological mechanisms will also increase our understanding of the preventive potential of these factors in a more personalized context. We are interested in known energy balance-related CRC risk or protective factors, including (abdominal) fatness as reflected by BMI and waist circumference, height^2,3^, physical activity^2,4^, and early life energy restriction^5^, and how the CRC risks associated with these factors may be modified by sirtuin 1 (SIRT1), an energy-sensing protein.

SIRT1 is a histone deacetylase that links the metabolic status of the cell to regulation of gene expression and numerous other processes with relevance to cancer, such as apoptosis, genetic stability, inflammation, immune response, and autophagy^6–9^. A large number of studies point towards a tumor suppressor role in cancer initiation for SIRT1^6^. However, the precise role of SIRT1 remains somewhat controversial as SIRT1 may have oncogenic activities as well^10^. Of particular interest to CRC and cancer in general is also SIRT1’s influence on glucose and lipid metabolism^6–9^, considering that altered cell metabolism is one of the a hallmarks of cancer^11^. Increases in SIRT1 expression level have been observed following nutrient deprivation^7,12^, possibly explaining in part the association between energy restriction and a decreased cancer risk^13^, whereas reduced expression levels have been associated with adiposity measures^14,15^, high glucose, insulin, insulin-like growth factor 1, and diabetes^6^. This suggests SIRT1 expression can be modified along the energy balance spectrum and concomitant diseases, with potential for CRC prevention when changing lifestyles that influence energy balance and CRC risk. Furthermore, SIRT1 might behold opportunities for more direct targeting in future CRC prevention, considering that emerging evidence suggests that aspirin^16^ has SIRT1-mediated anticancer effects.

SIRT1 expression level data could help substantiate human observational evidence for a role of SIRT1 in cancer but expression data are challenging to obtain in large population-based cohorts with long follow-up as expression levels are time-dependent and tissue-specific. Therefore, we used two *SIRT1* single nucleotide polymorphisms (SNPs) (rs10997870 and rs12778366) as time-independent indicators of SIRT1 involvement in carcinogenesis^17^. Firstly, we investigated these *SIRT1* variants in relation to CRC risk by sex and subsite using data from 20.3 years follow-up from the Netherlands Cohort Study (NLCS). The selected *SIRT1* variants covered 100% of the genetic variation in *SIRT1* at a 5% minor allele frequency or higher using aggressive tagging. Both variants have been reported to be expression quantitative trait loci (eQTL) for *SIRT1* in whole blood (rs10997870), esophagus mucosa (rs10997870 and rs12778366), and lung tissue (rs10997870) in the GTEx portal^18^. In all tissues, *SIRT1* rs10997870 major allele homozygotes showed higher SIRT1 expression levels as compared to heterozygotes and homozygotes for the minor allele, which showed the lowest SIRT1 expression level^18^. *SIRT1* rs12778366 heterozygotes and homozygotes for the minor allele (albeit there were only few of the latter) showed higher SIRT1 expression levels than homozygotes for the major allele in esophagus mucosa. Higher SIRT1 expression levels may seem favorable in terms of CRC risk given relationships with nutrient deprivation, adiposity, and other variables, as described above. However, caution is warranted when it comes to making assumptions about the association between SIRT1 expression levels and CRC risk, because dose-dependent effects of SIRT1 expression levels on cancer development have been found in mouse models, with different SIRT1 expression levels triggering different pathways^19^. Therefore, we hypothesized that the selected variants are associated with CRC risk, though the direction of the effect cannot be hypothesized. Secondly, we evaluated BMI, trouser/skirt size as a proxy for waist circumference, BMI at age 20, height, physical activity, and energy restriction in early life in relation to CRC risk by sex and subsite within genotype strata of *SIRT1* rs10997870 and rs12778366. We hypothesized that associations between energy balance-relatd factors and CRC risk differ (in strength) between strata of *SIRT1* genetic variants (effect modification), given SIRT1’s role in carcinogenesis and its role as an energy-sensing molecule. We expect CRC risk factor-associations, however, to be consistent with previous NLCS findings showing that a higher BMI and larger trouser/skirt size in men and tallness in women were associated with an increased CRC risk, particularly distal colon cancer risk; showing that a higher level of physical activity (occupational physical activity in men and non-occupational physical activity in women) was associated with a decreased CRC risk, particularly distal colon cancer risk; and showing that early life energy restriction in women was associated with a decreased CRC risk, particularly proximal colon and rectal cancer risk^3–5^.

## Methods

### Population and design

The NLCS is a nationwide cohort study in the Netherlands. In total, 340,439 individuals sampled from 204 Dutch municipalities were invited by mail to complete the baseline questionnaire and participate in the NLCS. The NLCS includes 120,852 men and women who all completed a questionnaire on diet and cancer and ~75% returned toenail clippings in 1986 when 55–69 years old^20^. The cohort is followed up using a case-cohort approach. A random subcohort of 5000 individuals was selected immediately after baseline. Exclusion of participants with a history of cancer, other than skin cancer, left 4774 subcohort members. We estimate the accumulated person-time at risk for the subcohort through linkage with the Central Bureau of Genealogy and municipal registries (>99.9% completeness) for information on vital status. We enumerate incident cancer cases through linkage with the population-based cancer registry, PALGA (the Netherlands pathology database), and the Central Bureau for Statistics (>96%completeness)^21,22^. The case-cohort design allows for the estimation of hazard ratios as would be done in a full cohort under the assumption that the fraction of the accumulated person-time at risk observed for exposed and unexposed individuals is equal. This can be assumed because the subcohort was selected independent of any exposure. The extra variance introduced by sampling the subcohort from the total cohort can be adjusted for using the robust variance estimator^23^. A detailed description of the NLCS is available in^20^. After 20.3 years of follow-up from September 1986 until the end of 2006, there were 3144 incident colon cancer cases (ICD-O-3 code C18) (among which 1623 incident proximal colon cancer cases (ICD-O-3 codes C18-C18.4) and 1430 incident distal colon cancer cases (ICD-O-3 codes C18.5-C18.7)), 427 incident rectosigmoid cancer cases (ICD-O-3 code C19), and 1026 incident rectal cancer cases (ICD-O-3 code C20), totaling to 4597 incident CRC cases.

### Ethics statement

The review boards of the TNO Nutrition and Food Research Institute (Zeist, the Netherlands) and Maastricht University (Maastricht, the Netherlands) approved the NLCS. Individuals invited to participate in the NLCS received an invitation letter with details on the study and they received the baseline questionnaire, which included an envelope for returning toenail clippings alongside with the questionnaire. Individuals agreed to participate in the NLCS by means of returning the baseline questionnaire (response rate 35.5%)^20^. All methods were performed in accordance with the relevant guidelines and regulations.

### SIRT1 genotyping

Toenail clippings are a valid and long-term DNA source, which can be stored without further treatment or climate control, for the genotyping of germline genetic variants^24,25^. DNA isolated from toenails according to an adapted protocol based on Cline *et al*.^26^ is stored at −30 °C at the BioBank Maastricht University Medical Center+ (Maastricht, the Netherlands). Genotyping of two *SIRT1* tagging SNPs (rs10997870, an intron variant, and rs12778366, an 5′ upstream gene variant) was done using the Agena BioScience MassARRAY® platform (Hamburg, Germany). Rs10997870 and rs12778366 are in low linkage disequilibrium (LD), r^2^ = 0.308, as based on the 1000 Genomes CEU population^27^. SNP call rates were 97%. The SNPs were part of a larger assay with 26 SNPs in total. A sample call rate of 95% or higher was present in 93.6% of samples from subcohort members and in 95.1% of samples, leaving 3550 subcohort members and 3293 CRC cases for further analyses.

### Questionnaire data

Questionnaire data were key-entered and processed in a manner blinded to subcohort or case status. Primary exposure variables related to energy balance used for modeling associations within genotype strata of *SIRT1* SNPs and to test interactions with *SIRT1* SNPs were derived from the baseline questionnaire. Self-reported information included weight at baseline (kg), weight at age 20 (kg), height (cm), trouser/skirt size (Dutch clothing sizing), non-occupational physical activity [sum measure of daily walking/cycling (min/day), weekly recreational walking/cycling, weekly gardening/doing odd jobs, and weekly sports/gymnastics (never, 1, 1–2, >2 hours/week), categorized as ≤30, 30–60, >60 min/day], and energy restriction during the Hunger Winter (1944–45), War Years (1940–44), and Economic Depression (1932–40). Weight and height were used to derive BMI in kg/m^2^ as a reflection of body fatness. Trouser/skirt size reflects waist circumference or abdominal fatness when adjusted for BMI. BMI measures were categorized in sex-specific tertiles based on the distribution in the subcohort and trouser/skirt size was dichotomized into below and median or above median sex-specific clothing sizes. Self-reports on weight and height have been shown valid measures in large cohort studies with >10 years follow-up^28,29^. Trouser/skirt size correlated with hip and waist circumferences in a subset of weight-stable NLCS men (r = 0.63 and 0.64, respectively) and women (r = 0.78 and 0.71, respectively) and was associated with endometrial and renal cancer risk in a fashion as would be expected for waist circumference^30^. Self-reported physical activity may not be without measurement error, but non-occupational physical activity as measured in our cohort was associated with a decreased risk of several cancers conform hypothesis^4,31–34^, suggesting adequate ranking of individuals in terms of physical activity level. Energy restriction was proxied by the place of residence during the Dutch Hunger Winter (non-western, Western rural, or Western city), the place of residence during the midpoint (1942) of the War Years (rural or urban), and the employment status of an individual’s father during the Economic Depression (employed or unemployed)^5,35^. It has been documented that lower energy intake was associated with an unemployed father during the Economic Depression (though calories remained sufficient but the variation in the food pattern was more limited), that food supplies deteriorated much faster in the cities than rural areas during the War Years, and that severe energy restriction was confined to the western (famine) cities (>40,000 inhabitants)^36^ during the Hunger Winter. The Hunger Winter lasted ~7 months with a low point from December 1944 until April 1945, and estimated caloric intake was between 400–800 kcal/day. Reports on this famine having effects on reproductive outcomes, birth weight, malformations, and perinatal mortality corroborate the severity of the energy restriction^37^. Eighty percent of female subcohort members in our cohort who, during follow-up, indicated that they had experienced severe hunger during the winter of 1944–45, reported to have lived in a western city^38^. We analyzed ER variables separately, since these describe different contrasts in different periods at young age, with subcohort members and CRC cases being between 0–23, 8–28, and 12–28 years old, respectively, during these consecutive periods.

The baseline questionnaire also provided information on covariates, including dietary factors, which were derived from a 150-item semi-quantitative food frequency questionnaire (FFQ) that was included in the baseline questionnaire. The FFQ assessed regular food intake in the preceding year and was found to rank individuals adequately according to dietary intake as compared with a 9-day dietary record^39^. It was also shown a good indicator of intake for at least 5 years^40^. Exclusion of individuals with incomplete/inconsistent questionnaires, on top of the genotyping-related exclusions, left 3337 subcohort members and 3112 CRC cases.

### Statistical analysis

We estimated sex- and subsite-specific hazard ratios (HRs) and corresponding 95% confidence intervals (CIs) for CRC according to *SIRT1* genotypes and categories of energy balance-related CRC risk factors (BMI in tertiles, trouser/skirt size (below and equal to or above median size), BMI at age 20 in tertiles, non-occupational physical activity (≤30, >30–60, >60 min/day), height in tertiles, and energy restriction during the Hunger Winter (non-western, western rural, western city), War Years (rural, urban), and Economic Depression (father unemployed, father employed)) within rs12778366 and rs10997870 genotype strata. *SIRT1* SNP models were analyzed under the (conservative) assumption of a co-dominant inheritance mode, adjusting for age. In addition, we ran an analysis in which we assumed an additive inheritance mode to explore the per additional minor allele-risk association. Models for energy balance-related CRC risk factors stratified by rs12778366 and rs10997870 genotypes were adjusted for potential confounders for the risk factor-CRC association. Genotype strata were defined assuming a dominant inheritance mode for reasons of power. In accordance with the literature on convincing or probable CRC risk factors^41^ and previous analyses within the NLCS^3–5,42^, covariate adjustment was made for age (years), first-degree family history of colorectal cancer (yes/no), smoking status (never, ex, current), and intake of alcohol (0, 0.1–29, ≥30 g/d), meat (g/d), processed meat (g/d), and total energy (kcal/d). In addition, all models, except models for physical activity, were adjusted for physical activity (≤30, >30–60, >60 min/day) and all models, except models for BMI and physical activity, were adjusted for BMI (kg/m^2^). Analyses were performed using R statistical software (version 3.2.2). Cox models (*coxph*, survival package) were adjusted for the additional variance introduced by sampling the subcohort from the total cohort by estimating standard errors using the robust Huber-White sandwich estimator^23^ [i.e. entering the participant identification number as cluster term in the model]. We checked potential violations of the proportional hazards assumption by plotting the scaled Schoenfeld residuals against time and violations appeared minimal (*cox.zph*, survival package). Multiplicative interactions were tested with the Wald test (*wald.test*, aod package). Statistical significance was indicated by a *P-*value < 0.05 for two-sided testing. False discovery rate-adjusted *P*-values across men and women were calculated according to the method of Benjamini and Hochberg for Wald *P*-values for interactions^43^. The FDR adjustment entailed ranking *P*-values in ascending order and multiplying a predefined FDR threshold (0.20^44^) with the inverse of the rank order over the total number of *P*-values considered to be part of the multiple testing. If the original *P*-value was below 0.05 and below the FDR-adjusted *P*-value, we considered the interaction statistically significant.

## Results

A flow chart of subcohort members and CRC cases with available genotyping information and information on energy balance-related factors is shown in Supplemental Fig. 1. *SIRT1* rs10997870 TT, TG, and GG genotype frequencies did not differ between subcohort members and CRC cases (40.0, 47.2, and 12.8 percent in the male subcohort versus 41.4, 45.6, and 13.0 percent in male CRC cases; and 38.4, 47.6 and 14.0 percent in the female subcohort versus 39.5, 47.0, and 13.5 percent in female CRC cases). Comparison of *SIRT1* rs12778366 TT, TC, and CC genotype frequencies between subcohort members and CRC cases showed that slightly more subcohort members than CRC cases carried one or two copies of the minor allele (72.8, 25.0, and 2.2 percent in the male subcohort versus 74.7, 23.6, and 1.7 percent in male CRC cases; and 71.4, 26.2, and 2.4 percent in the female subcohort versus 74.3, 24.3, and 1.4 in female CRC cases). Baseline characteristics of subcohort members were fairly comparable across *SIRT1* genotype strata defined according to a dominant model (Table 1).

**Table 1:** Baseline characteristics of men and women in the Netherlands Cohort Study by *SIRT1* rs10997870 and rs12778366 genotypes (dominant model).

Characteristic	Male subcohort				Female subcohort			
	rs10997870 TT	rs10997870 TG/GG	rs12778366 TT	rs12778366 TC/CC	rs10997870 TT	rs10997870 TG/GG	rs12778366 TT	rs12778366 TC/CC
N	690	1034	1255	469	586	941	1090	436
BMI, mean (SD) kg/m^2^	24.9 (2.6)	25.0 (2.5)	25.0 (2.6)	24.8 (2.5)	24.9 (3.5)	25.1 (3.6)	25.1 (3.7)	24.9 (3.4)
Waist circumference, %								
<median Dutch clothing size	33.9	34.4	33.6	35.8	44.9	42.1	43.3	42.9
≥median	57.0	56.5	57.5	54.4	54.1	56.1	55.4	55.0
BMI at 20 years, mean (SD) kg/m^2^	21.7 (2.4)	21.7 (2.4)	21.8 (2.4)	21.7 (2.4)	21.4 (2.7)	21.4 (2.6)	21.4 (2.6)	21.2 (2.7)
Height, mean (SD) cm	176.3 (6.7)	176.6 (6.7)	176.4 (6.6)	176.7 (7.1)	165.3 (5.9)	165.1 (6.3)	165.1 (6.2)	165.3 (6.1)
Non-occupational physical activity, %								
≤30 min/day	15.1	18.1	15.9	19.4	18.9	24.2	21.2	24.8
>30–60	31.4	31.6	32.5	29.0	32.2	32.6	32.3	32.8
>60	52.2	49.5	50.3	51.4	47.4	42.2	45.4	41.3
Energy restriction during the Hunger Winter, %								
Non-western place of residence	45.7	51.4	48.6	50.5	51.2	53.2	53.2	50.5
Western rural place of residence	13.6	11.7	12.0	13.6	14.3	14.9	14.8	14.4
Western city place of residence	20.4	19.6	20.3	19.4	29.0	25.6	26.5	28.0
Energy restriction during the War Years, %								
Rural place of residence	35.9	36.9	36.2	37.5	35.7	34.0	35.7	32.1
Urban place of residence	39.1	35.8	37.8	35.2	41.1	37.8	38.3	41.3
Energy restriction during the Economic Depression, %								
Father employed	86.2	85.0	85.4	85.7	86.5	83.8	85.3	83.7
Father unemployed	9.4	10.0	9.6	10.0	10.1	10.1	9.9	10.6
Age, years	61.4 (4.2)	61.3 (4.2)	61.4 (4.2)	61.3 (4.3)	61.3 (4.2)	61.5 (4.3)	61.4 (4.2)	61.6 (4.3)
Family history of CRC, %								
No	93.5	95.2	94.1	95.5	93.7	94.3	93.7	95.0
Yes	6.5	4.8	5.9	4.5	6.3	5.7	6.3	5.0
Smoking status, %								
Never	12.9	12.9	13.1	12.2	56.8	58.4	57.2	59.4
Ex	52.9	53.1	52.7	53.9	24.4	19.4	22.2	19.3
Current	34.2	34.0	34.2	33.9	18.8	22.1	20.6	21.3
Alcohol intake, %								
0 g/d	15.8	12.7	14.3	12.8	30.5	32.8	31.9	32.1
0.1–29	71.1	71.3	71.9	69.5	65.5	63.9	64.5	64.4
≥30	13.0	16.1	13.8	17.7	3.9	3.3	3.6	3.4
Processed meat intake, mean (SD) g/d	16.3 (16.8)	17.4 (18.1)	16.9 (18.1)	17.2 (16.3)	10.7 (12.1)	11.0 (12.3)	10.9 (12.3)	11.0 (12.3)
Meat intake, mean (SD) g/d	106.4 (42.1)	104.7 (43.3)	105.6 (42.6)	104.8 (43.4)	92.8 (39.6)	93.0 (40.9)	93.0 (40.8)	92.6 (39.4)
Total energy intake, mean (SD) kcal/day	2155 (488)	2155 (505)	2150 (501)	2168 (493)	1699 (404)	1685 (389)	1696 (398)	1675 (384)

Note: percentages may not add up to 100% because of missing values on variables. Abbreviations: CRC, colorectal cancer; SD, standard deviation.

Table 2 shows *SIRT1* variants in relation to CRC risk by sex and subsite after 20.3 years of follow-up. *SIRT1* rs10997870 was not associated with any of the CRC endpoints considered in men and women in both co-dominant and additive models. *SIRT1* rs12778366 was also not associated with any of the CRC endpoints considered in men in both co-dominant and additive models. Comparison of the rs12778366 CC versus TT genotype yielded decreased CRC and colon cancer risks in women (HR for CRC = 0.53, 95% confidence interval: 0.30–0.94; HR for colon cancer = 0.53, 95% CI: 0.29–1.00). *SIRT1* rs12778366 was not statistically associated with the risk of proximal colon and rectal cancer in women, though hazard ratios were also below one. The rs12778366 CC versus TT genotype could not be compared in terms of distal colon cancer risk in women, because there were only two female distal colon cancer cases with the CC genotype. Analyses per additional minor allele for rs12778366 furthermore indicated inverse associations with all endpoints in women. Hazard ratios for the per minor allele model were less strongly decreased than when comparing rs12778366 CC with TT genotypes and only statistically significant in relation to CRC (HR = 0.84, 95% CI: 0.73–0.97).

**Table 2:** *SIRT1* tagging single nucleotide polymorphisms in relation to colorectal cancer risk overall and by subsite in men and women from the Netherlands Cohort Study (20.3 years follow-up).

Variant	Comparison	Endpoint	Men				Women			
			PT at risk	N cases	HR^a^	(95% CI)	PT at risk	N cases	HR^a^	(95% CI)
rs10997870	TG vs. TT	Colorectum	13446/11698	868/766	0.99	(0.86,1.14)	14230/11943	658/561	0.98	(0.84,1.14)
	GG vs. TT		3697/11698	248/766	1.03	(0.83,1.28)	4224/11943	192/561	0.96	(0.77,1.20)
	Per minor allele		28841	1882	1.01	(0.91,1.11)	30397	1411	0.98	(0.88,1.09)
rs10997870	TG vs. TT	Colon	13446/11698	556/491	0.99	(0.84,1.17)	14230/11943	488/401	1.01	(0.85,1.20)
	GG vs. TT		3697/11698	170/491	1.10	(0.87,1.40)	4224/11943	155/401	1.08	(0.85,1.38)
	Per minor allele		28841	1217	1.03	(0.92,1.16)	30397	1044	1.03	(0.92,1.16)
rs10997870	TG vs. TT	Proximal colon	13446/11698	265/224	1.04	(0.84,1.28)	14230/11943	277/241	0.95	(0.78,1.17)
	GG vs. TT		3697/11698	76/224	1.08	(0.79,1.47)	4224/11943	99/241	1.15	(0.87,1.52)
	Per minor allele		28841	565	1.04	(0.90,1.20)	30397	617	1.04	(0.91,1.20)
rs10997870	TG vs. TT	Distal colon	13446/11698	271/255	0.93	(0.76,1.14)	14230/11943	195/155	1.05	(0.83,1.33)
	GG vs. TT		3697/11698	92/255	1.15	(0.86,1.53)	4224/11943	52/155	0.94	(0.67,1.34)
	Per minor allele		28841	618	1.03	(0.90,1.19)	30397	402	0.99	(0.85,1.16)
rs10997870	TG vs. TT	Rectum	13446/11698	219/208	0.92	(0.74,1.14)	14230/11943	122/110	0.93	(0.70,1.22)
	GG vs. TT		3697/11698	57/208	0.87	(0.62,1.21)	4224/11943	28/110	0.72	(0.46,1.11)
	Per minor allele		28841	484	0.93	(0.80,1.08)	30397	260	0.87	(0.72,1.06)
rs12778366	TC vs. TT	Colorectum	7046/21086	451/1398	0.98	(0.84,1.15)	7904/21761	340/1052	0.88	(0.74,1.03)
	CC vs. TT		709/21086	33/1398	0.68	(0.42,1.10)	719/21761	19/1052	0.53	(0.30,0.94)
	Per minor allele		28841	1882	0.93	(0.82,1.07)	30384	1411	0.84	(0.73,0.97)
rs12778366	TC vs. TT	Colon	7046/21086	294/898	1.00	(0.84,1.19)	7904/21761	258/772	0.90	(0.76,1.08)
	CC vs. TT		709/21086	25/898	0.80	(0.48,1.34)	719/21761	14/772	0.53	(0.29,1.00)
	Per minor allele		28841	1217	0.97	(0.83,1.12)	30384	1044	0.86	(0.74,1.01)
rs12778366	TC vs. TT	Proximal colon	7046/21086	134/417	0.99	(0.78,1.24)	7904/21761	146/460	0.85	(0.69,1.06)
	CC vs. TT		709/21086	14/417	0.95	(0.50,1.80)	719/21761	11/460	0.70	(0.35,1.39)
	Per minor allele		28841	565	0.98	(0.81,1.19)	30384	617	0.85	(0.70,1.03)
rs12778366	TC vs. TT	Distal colon	7046/21086	152/455	1.01	(0.82,1.26)	7904/21761	101/299	0.92	(0.72,1.19)
	CC vs. TT		709/21086	11/455	0.70	(0.36,1.37)	719/21761	2/299	^b^	
	Per minor allele		28841	618	0.96	(0.80,1.15)	30384	402	0.82	(0.66,1.02)
rs12778366	TC vs. TT	Rectum	7046/21086	110/369	0.90	(0.71,1.15)	7904/21761	62/193	0.87	(0.64,1.19)
	CC vs. TT		709/21086	5/369	0.40	(0.15,1.02)	719/21761	5/193	0.76	(0.30,1.97)
	Per minor allele		28841	484	0.83	(0.68,1.02)	30384	260	0.87	(0.67,1.14)

^a^Age-adjusted hazard ratio for colorectal cancer.

^b^Left blank because this cell contained fewer than 5 cancer cases.

Table 3 and the Supplemental Tables 1–4 show the results of energy balance-related CRC risk factors in relation to the risk of CRC overall and by subsite in men and women stratified by *SIRT1* genotypes according to a dominant inheritance model. Table 3 shows that, consistent with expectations, positive associations were present between BMI and CRC risk in men, trouser/skirt size and CRC risk in men, and height and CRC risk in men and women, while inverse associations were present between non-occupational physical activity and CRC risk in women, and that associations were present in either one or both genotype strata for rs10997870 and rs12778366. No statistically significant interaction was observed between these exposures and the variants. A pattern was lacking as regards to which genotype stratum showed associations. Table 3 also shows that *SIRT1* rs10997870 significantly interacted with BMI at age 20 in men and BMI in women in relation to CRC risk. Male major allele (TT) carriers in the middle versus those in the lowest BMI tertile for BMI at age 20 had a significantly decreased CRC risk (HR = 0.67, 95% CI: 0.49, 0.91). No statistically significant associations were observed between BMI and CRC risk in women in either major (TT) or minor allele (TG/GG) carriers, although HRs were borderline statistically significantly decreased when comparing the middle BMI tertile with the lowest in minor allele carriers (rs10997870 TG/GG: HR = 0.79, 95% CI: 0.62–1.01; rs12778366 TC/CC: HR = 0.74, 95% CI: 0.51–1.07).

**Table 3:** Exposures related to energy balance in relation to colorectal cancer risk in men and women stratified by genotype strata (dominant model) of SIRT1 variants in the Netherlands Cohort Study (20.3 years of follow-up).

		Men										Women									
		BMI										BMI									
		T1 sex-specific (13.5–23.9 kg/m^2^)			T2 sex-specific (23.8–25.9 kg/m^2^)			T3 sex-specific (25.8–41.3 kg/m^2^)			*P* for interaction	T1 sex-specific (14.5–23.5 kg/m^2^)			T2 sex-specific (23.4–26.2 kg/m^2^)			T3 sex-specific (26.1–42.2 kg/m^2^)			*P* for interaction
		N cases/PT at risk	HR^a^	(95% CI)	N cases/PT at risk	HR^a^	(95% CI)	N cases/PT at risk	HR^a^	(95% CI)		N cases/PT at risk	HR^a^	(95% CI)	N cases/PT at risk	HR^a^	(95% CI)	N cases/PT at risk	HR^a^	(95% CI)	
rs10997870	TT	220/3472	1	(ref.)	214/3641	0.86	(0.64,1.14)	276/3454	1.15	(0.87,1.54)		161/3796	1	(ref.)	180/3045	1.29	(0.95,1.76)	142/3140	1.02	(0.74,1.40)	
	TG/GG	296/5466	1	(ref.)	360/5512	1.14	(0.90,1.43)	352/4670	1.36	(1.07,1.72)	0.37	268/5240	1	(ref.)	230/5675	0.79	(0.62,1.01)	236/5170	0.89	(0.69,1.15)	0.04^b^
rs12778366	TT	377/6169	1	(ref.)	420/6782	0.97	(0.78,1.19)	488/6247	1.20	(0.97,1.48)		309/6608	1	(ref.)	317/5989	1.09	(0.87,1.36)	279/5974	0.95	(0.75,1.20)	
	TC/CC	139/2769	1	(ref.)	154/2371	1.17	(0.82,1.66)	140/1877	1.47	(1.01,2.13)	0.53	120/2427	1	(ref.)	93/2731	0.74	(0.51,1.07)	99/2322	0.97	(0.65,1.45)	0.10
		**Trouser/skirt size**										**Trouser/skirt size**									
		**<median**			**≥median**						***P*** **for interaction**	**<median**			**≥median**						***P*** **for interaction**
		**N cases/ PT at risk**	**HR** ^**a**^	**(95% CI)**	**N cases/ PT at risk**	**HR** ^**a**^	**(95% CI)**					**N cases/ PT at risk**	**HR** ^**a**^	**(95% CI)**	**N cases/ PT at risk**	**HR** ^**a**^	**(95% CI)**				
rs10997870	TT	219/3651	1	(ref.)	435/6091	0.99	(0.75,1.32)					209/4593	1	(ref.)	266/5320	1.16	(0.83,1.60)				
	TG/GG	297/5592	1	(ref.)	621/8745	1.31	(1.04,1.64)				0.30	313/6850	1	(ref.)	415/8964	1.00	(0.77,1.30)				0.55
rs12778366	TT	384/6539	1	(ref.)	798/11144	1.12	(0.91,1.37)					384/8290	1	(ref.)	508/10085	1.09	(0.86,1.39)				
	TC/CC	132/2704	1	(ref.)	258/3692	1.33	(0.94,1.87)				0.25	138/3153	1	(ref.)	173/4185	0.97	(0.65,1.43)				0.38
		**BMI @ 20 years**										**BMI @ 20 years**									
		**T1 sex-specific (11.3–20.8 kg/m** ^**2**^ **)**			**T2 sex-specific (20.7–22.7 kg/m** ^**2**^ **)**			**T3 sex-specific (22.6–33.1 kg.m** ^**2**^ **)**			***P*** **for interaction**	**T1 sex-specific (11.2–20.3 kg/m** ^**2**^ **)**			**T2 sex-specific (20.2–22.5 kg/m** ^**2**^ **)**			**T3 sex-specific (22.4–46.9 kg/m** ^**2**^ **)**			***P*** **for interaction**
		**N cases/ PT at risk**	**HR** ^**a**^	**(95% CI)**	**N cases/ PT at risk**	**HR** ^**a**^	**(95% CI)**	**N cases/ PT at risk**	**HR** ^**a**^	**(95% CI)**		**N cases/ PT at risk**	**HR** ^**a**^	**(95% CI)**	**N cases/ PT at risk**	**HR** ^**a**^	**(95% CI)**	**N cases/ PT at risk**	**HR** ^**a**^	**(95% CI)**	
rs10997870	TT	222/2883	1	(ref.)	168/2946	0.67	(0.49,0.91)	201/2616	0.88	(0.63,1.22)		144/3006	1	(ref.)	162/3265	1.07	(0.76,1.49)	146/2892	1.16	(0.80,1.69)	
	TG/GG	264/4459	1	(ref.)	282/4142	1.18	(0.91,1.54)	280/4141	1.09	(0.84,1.43)	0.03^b^	224/4993	1	(ref.)	224/4867	1.05	(0.81,1.37)	212/4723	1.05	(0.79,1.39)	0.98
rs12778366	TT	363/5144	1	(ref.)	343/5209	0.93	(0.74,1.18)	358/4952	0.99	(0.78,1.26)		274/5528	1	(ref.)	287/5986	1.00	(0.79,1.27)	270/5474	1.04	(0.79,1.35)	
	TC/CC	123/2197	1	(ref.)	107/1880	1.00	(0.67,1.48)	123/1805	1.07	(0.71,1.60)	0.82	94/2472	1	(ref.)	99/2146	1.32	(0.87,2.00)	88/2127	1.21	(0.79,1.84)	0.72
		**Non-occupational physical activity**										**Non-occupational physical activity**									
		**≤30 min/day**			**>30–60 min/day**			**>60 min/day**			***P*** **for interaction**	**≤30 min/day**			**>30–60 min/day**			**>60 min/day**			***P*** **for interaction**
		**N cases/PT at risk**	**HR** ^**a**^	**(95% CI)**	**N cases/PT at risk**	**HR** ^**a**^	**(95% CI)**	**N cases/ PT at risk**	**HR** ^**a**^	**(95% CI)**		**N cases/PT at risk**	**HR** ^**a**^	**(95% CI)**	**N cases/PT at risk**	**HR** ^**a**^	**(95% CI)**	**N cases/PT at risk**	**HR** ^**a**^	**(95% CI)**	
rs10997870	TT	123/1540	1	(ref.)	220/3442	0.80	(0.57,1.13)	367/5584	0.81	(0.58,1.12)		122/1752	1	(ref.)	156/3272	0.69	(0.48,1.00)	205/4957	0.61	(0.43,0.86)	
	TG/GG	151/2559	1	(ref.)	295/5145	0.92	(0.69,1.23)	562/7945	1.15	(0.88,1.50)	0.15	191/3843	1	(ref.)	233/5298	0.90	(0.69,1.18)	310/6943	0.90	(0.70,1.16)	0.17
rs12778366	TT	214/2873	1	(ref.)	390/6509	0.77	(0.60,1.00)	681/9816	0.90	(0.71,1.14)		235/3761	1	(ref.)	283/6103	0.76	(0.59,0.98)	387/8707	0.72	(0.57,0.91)	
	TC/CC	60/1226	1	(ref.)	125/2078	1.20	(0.76,1.88)	248/3712	1.33	(0.88,2.00)	0.18	78/1834	1	(ref.)	106/2453	1.04	(0.70,1.57)	128/3193	0.89	(0.60,1.32)	0.42
		**Height**										**Height**									
		**T1 sex-specific (147–173 cm)**			**T2 sex-specific (174–179 cm)**			**T3 sex-specific (180–202 cm)**			***P*** **for interaction**	**T1 sex-specific (140–163 cm)**			**T2 sex-specific (164–168 cm)**			**T3 sex-specific (169–200 cm)**			***P*** **for interaction**
		**N cases/ PT at risk**	**HR** ^**a**^	**(95% CI)**	**N cases/ PT at risk**	**HR** ^**a**^	**(95% CI)**	**N cases/ PT at risk**	**HR** ^**a**^	**(95% CI)**		**N cases/ PT at risk**	**HR** ^**a**^	**(95% CI)**	**N cases/ PT at risk**	**HR** ^**a**^	**(95% CI)**	**N cases/ PT at risk**	**HR** ^**a**^	**(95% CI)**	
rs10997870	TT	238/3843	1	(ref.)	240/3411	1.15	(0.87,1.51)	232/3313	1.20	(0.91,1.59)		167/3808	1	(ref.)	162/3354	1.24	(0.90,1.70)	154/2818	1.34	(0.96,1.87)	
	TG/GG	297/5261	1	(ref.)	336/5055	1.25	(0.99,1.58)	375/5332	1.37	(1.08,1.73)	0.76	230/6212	1	(ref.)	275/5468	1.37	(1.07,1.74)	229/4404	1.44	(1.11,1.87)	0.76
rs12778366	TT	408/6730	1	(ref.)	438/6281	1.17	(0.95,1.44)	439/6187	1.23	(1.00,1.51)		296/7193	1	(ref.)	320/6286	1.30	(1.04,1.63)	289/5092	1.46	(1.15,1.86)	
	TC/CC	127/2374	1	(ref.)	138/2185	1.30	(0.91,1.87)	168/2458	1.46	(1.01,2.09)	0.91	101/2827	1	(ref.)	117/2523	1.32	(0.91,1.92)	94/2130	1.20	(0.81,1.79)	0.76
		**Residence Hunger Winter**										**Residence Hunger Winter**									
		**Non-Western area**			**Western rural area**			**Western city**			***P*** **for interaction**	**Non-Western area**			**Western rural area**			**Western city**			***P*** **for interaction**
		**N cases/PT at risk**	**HR** ^**a**^	**(95% CI)**	**N cases/PT at risk**	**HR** ^**a**^	**(95% CI)**	**N cases/PT at risk**	**HR** ^**a**^	**(95% CI)**		**N cases/PT at risk**	**HR** ^**a**^	**(95% CI)**	**N cases/PT at risk**	**HR** ^**a**^	**(95% CI)**	**N cases/PT at risk**	**HR** ^**a**^	**(95% CI)**	
rs10997870	TT	393/4973	1	(ref.)	75/1402	0.75	(0.52,1.07)	126/2177	0.74	(0.54,1.01)		247/5204	1	(ref.)	80/1372	1.35	(0.92,1.98)	130/2914	0.96	(0.70,1.31)	
	TG/GG	505/7945	1	(ref.)	141/1901	1.27	(0.94,1.70)	201/3242	0.96	(0.75,1.23)	0.06	373/8610	1	(ref.)	113/2315	1.17	(0.87,1.58)	206/4172	1.17	(0.92,1.49)	0.36
rs12778366	TT	697/9391	1	(ref.)	142/2267	0.92	(0.70,1.21)	225/3968	0.76	(0.60,0.95)		466/9984	1	(ref.)	144/2662	1.20	(0.92,1.59)	245/4940	1.08	(0.86,1.35)	
	TC/CC	201/3527	1	(ref.)	74/1036	1.31	(0.85,2.01)	102/1451	1.18	(0.81,1.73)	0.08	154/3816	1	(ref.)	49/1025	1.24	(0.78,1.98)	91/2145	1.09	(0.76,1.55)	0.97
		**Residence World War 2**										**Residence World War 2**									
		**Rural area**			**Urban area**						***P*** **for interaction**	**Rural area**			**Urban area**						***P*** **for interaction**
		**N cases/ PT at risk**	**HR** ^**a**^	**(95% CI)**	**N cases/ PT at risk**	**HR** ^**a**^	**(95% CI)**					**N cases/ PT at risk**	**HR** ^**a**^	**(95% CI)**	**N cases/ PT at risk**	**HR** ^**a**^	**(95% CI)**				
rs10997870	TT	252/3885	1	(ref.)	288/4116	1.14	(0.87,1.49)					191/3632	1	(ref.)	181/4124	0.82	(0.61,1.12)				
	TG/GG	389/5734	1	(ref.)	367/5679	0.95	(0.76,1.18)				0.48	263/5466	1	(ref.)	294/6157	1.00	(0.78,1.27)				0.28
rs12778366	TT	472/7058	1	(ref.)	485/7304	0.99	(0.82,1.21)					350/6645	1	(ref.)	345/7228	0.91	(0.73,1.13)				
	TC/CC	169/2560	1	(ref.)	170/2491	1.07	(0.76,1.51)				0.83	104/2453	1	(ref.)	130/3052	1.02	(0.71,1.48)				0.59
		**Employment status father Economic Depression**										**Employment status father Economic Depression**									
		**Employed**			**Unemployed**						***P*** **for interaction**	**Employed**			**Unemployed**						***P*** **for interaction**
		**N cases/ PT at risk**	**HR** ^**a**^	**(95% CI)**	**N cases/ PT at risk**	**HR** ^**a**^	**(95% CI)**					**N cases/ PT at risk**	**HR** ^**a**^	**(95% CI)**	**N cases/ PT at risk**	**HR** ^**a**^	**(95% CI)**				
rs10997870	TT	602/9230	1	(ref.)	80/927	1.35	(0.93,1.97)					417/8710	1	(ref.)	46/994	0.96	(0.61,1.51)				
	TG/GG	873/13333	1	(ref.)	103/1644	0.89	(0.65,1.22)				0.09	632/13459	1	(ref.)	70/1662	0.88	(0.62,1.23)				0.74
rs12778366	TT	1099/16567	1	(ref.)	137/1842	1.07	(0.81,1.41)					782/15873	1	(ref.)	85/1853	0.91	(0.66,1.25)				
	TC/CC	376/5996	1	(ref.)	46/728	1.03	(0.64,1.66)				0.69	267/6282	1	(ref.)	31/803	0.93	(0.56,1.54)				0.94

Abbreviations: BMI, body mass index; CI, confidence interval; HR, hazard ratio; N, number of; PT, person-time; ref., reference; T1–3, tertile 1–3.

^a^Adjusted for age (years), first-degree family history of colorectal cancer (yes/no), smoking status (never, ex, current), alcohol intake (0, 0.1–29, ≥30 g/d), meat intake (g/d), processed meat intake (g/d), and total energy intake (kcal/d); all models except models for physical activity were additionally adjusted for physical activity (≤30, >30–60, >60 min/day); all models, except models for BMI and physical activity, were additionally adjusted for baseline BMI (kg/m^2^).

^b^Remained significant after comparison with the *p*-value adjusted for the Benjamini and Hochberg false discovery rate, setting the false discovery threshold at 0.20.

The stratified results in relation to colon, proximal colon, distal colon, and rectal cancer risks were generally similar to those for CRC (Supplemental Tables 1–4). Therefore, in this paragraph, we only describe the additionally observed statistically significant interactions. In relation to the risk of proximal colon cancer (Supplemental Table 2), there was a statistically significant interaction between rs12778366 and non-occupational physical activity in men, with decreased risks observed for higher physical activity levels as compared to the lowest (<30 min/day). In relation to distal colon cancer risk (Supplemental Table 3), we observed a statistically significant interaction between Hunger Winter exposure and rs12778366 in men, with increased risks observed for Hunger Winter exposure among minor allele (TC/CC) carriers. In relation to rectal cancer risk (Supplemental Table 4), there was a statistically significant interaction between rs1099787 and the employment status of an individual’s father during the Economic Depression as proxy for early life energy restriction, but there was no significant association within the genotype strata. Overall, again, there was little consistency regarding which genotype stratum showed associations. Some statistically significant assocations were consistent with expectation, while others were contrary to expectation. Of note was that height was consistently positively associated with colon cancer risk in men and colon and rectal cancer risk in women, independent of genotype stratum.

## Discussion

This study is one of few studies showing epidemiological data on associations between *SIRT1* tagging SNPs and cancer risk. The NLCS is, to the best of our knowledge, the only study that investigated *SIRT1* variants in relation to CRC risk, that studied associations by sex and colorectal subsite, and that investigated possible interactions of *SIRT1* variants with energy balance-related CRC risk factors. As regards the two *SIRT1* variants investigated, i.e. rs10997870 and rs12778366, rs12778366 female homozygous minor allele carriers had decreased CRC and colon cancer risks as compared to homozygous major allele carriers. *SIRT1* rs10997870 was not associated with CRC risk in men and women in this study. We will discuss these findings first.

Previous studies on *SIRT1* genetic variants in relation to cancer risk are scarce and were conducted in specific populations. A study in uranium miners with radon exposure found *SIRT1* rs7097008 to be associated with the risk of squamous cell carcinoma of the lung, as one of several variants tested, including rs10997870 and rs12778366^45^. *SIRT1* rs7097008 is a perfect proxy of rs3758391 (1000 Genomes CEU population: r^2^ = 1 and D’ = 1) and both are in high LD with rs10997870 (1000 Genomes CEU population: r^2^ = 0.892, D’ = 1)^27^. *SIRT1* rs3758391 was reported to be more common in Egyptian breast cancer patients than controls, as was rs12778366^46^. Rs12778366 was also one of the *SIRT1* variants analyzed in a Chinese study on lung cancer risk, but this study showed no significant associations^47^. Lung cancer differs etiologically from CRC, making a comparison with these results more difficult, but (postmenopausal) breast cancer shares several risk factors with CRC, including body fatness^41^. The results from the Egyptian study on breast cancer are in apparent accordance with our results, as this study showed homozygous major allele carriers to be more common among breast cancer patients than controls, while we observed female homozygous minor allele carriers to be at a decreased CRC risk as compared to homozygous major allele carriers.

As for the analyses on modification by *SIRT1* rs10997870 and rs12778366 of associations between energy balance-related factors and CRC risk, multiplicative interactions were observed between these *SIRT1* variants and several of the energy balance-related CRC risk factors considered. However, several of the associations observed within genotype strata in the presence of a significant interaction were opposite to hypothesis as based on current understanding of risk factors through literature. Therefore, caution is warranted for chance or spurious findings. Noticeably and consistent with literature and previous findings in the NLCS^2,3^, height, on the other hand, was a consistent colon cancer risk factor in men and a colon and rectal cancer risk factor in women; that is, this was observed independent of rs10997870 and rs12778366 genotype strata. Although height is reported as a risk factor for CRC in men in the literature^41^, there was no apparent association between height and CRC risk (or cancer risk at any colorectal subsite) in men within the NLCS when using data from 16.3 years of follow-up^3^. An association between height and colon cancer in men appeared only after *SIRT1* variation was taken into account in this study, and after variation in the insulin-like growth factor pathway was taken into account^42^ and in relation to the risk of *BRAF* mutated and MSI colorectal tumors in previous studies^48^.

It is unclear why height in earlier analyses in the NLCS after 16.3 years of follow-up was not found as a CRC risk factor in men but only in women. Current results stress the importance of taking biological mechanisms into account and the potential for masked associations when analyses are performed overall. Height is a marker of increased cell growth and proliferation and can be influenced by childhood exposures such as energy restriction^49^. SIRT1 acts as an energy-sensing protein influencing growth processes, particularly in response to energy restriction^13^. Both height and SIRT1 have been associated with human longevity, possibly through influencing cancer risks. Height has been associated with an increased risk of several types of cancer^41^ and increased cancer and all-cause mortality rates^50^. Decreased expression of SIRT1 in peripheral blood mononuclear cells has been associated with older age^51^ and minor allele carriers of *SIRT1* rs12778366, which decreased colon cancer risk in women in our study, were found to be at a significantly reduced mortality risk^52^. Collectively, these findings point to the importance of mechanisms regulating cell metabolism and growth from early life onwards in relation to CRC and aging in general.

To address the statistically significant associations between energy balance-related CRC risk factors and CRC risk within *SIRT1* rs10997870 and rs12778366 genotype strata that were not consistent with current understanding of CRC risk factors, some speculative explanations are discussed. The observed inverse association between BMI and CRC risk in women might be possible if the protective effects of estrogens produced in adipose tissue in postmenopausal women^53^ were not offset by an unhealthy metabolic state. The observed inverse association between BMI at age 20 and CRC risk in men could be due to an unfortunate reference category, which may have included unhealthy underweight men. Perhaps stratification on *SIRT1* genotypes tapped into these groups by chance, although this does not seem a more likely explanation than these findings being spurious. Alternatively, individuals with an intermediate BMI might have had a more stable weight with less weight gain during life and follow-up, as compared to individuals in the lowest BMI tertile. It has been shown that adult weight gain is associated with colon cancer and especially harmful in this respect are associated abdominal fatness and metabolic dysfunction^54^. Although this might have played a role, further research is needed to elucidate a potential modifying effect of *SIRT1* variation on energy balance-related CRC risk factors.

Strengths of this study include its prospective character and long follow-up with a large number of CRC cases, which minimizes the chance of selection and recall bias. A limitation of this study was the single baseline measurement of exposures, which may not have been representative for energy balance-related exposures over a follow-up of 20.3 years. If changes in BMI over follow-up affected associations, it may not be surprising that height, which is not modifiable, was consistently associated with CRC risk in the direction as expected.

In conclusion, *SIRT1* rs12778366 influenced colon cancer risk in women, which supports that SIRT1, an energy-sensing molecule, is involved in colon cancer development in women. No conclusions could be made regarding a modifying effect of *SIRT1* variants on associations between energy balance-related factors and CRC risk.

## Electronic supplementary material

Supplemental Figure 1
