# Supplementary material for: Sirtuin 1 genetic variation, energy balance and colorectal cancer risk by sex and subsite in the Netherlands Cohort Study
Source: Sci Rep. 2018 Nov 8;8:16540. doi: 10.1038/s41598-018-34728-6 (PMC6224413; doi:10.1038/s41598-018-34728-6)
Supplement: Supplementary file 1 — Supplemental Figure 1 [file 41598_2018_34728_MOESM1_ESM.pdf]

Sirtuin 1 genetic variation, energy balance and colorectal cancer risk by sex and subsite in the Netherlands Cohort Study

Simons CCJM[1], Schouten LJ[1], Godschalk RW[2], van Schooten FJ[2], van den Brandt PA[1,3], Weijenberg MP[1].

[1] Department of Epidemiology, GROW – School for Oncology and Developmental Biology, Maastricht University, Maastricht, The Netherlands

[2] Department of Toxicology & Pharmacology, NUTRIM School of Nutrition and Translational Research in Metabolism, Maastricht University, Maastricht, The Netherlands

[3] Department of Epidemiology, CAPHRI- School for Public Health and Primary Care, Maastricht University Medical Center+, Maastricht, The Netherlands

Corresponding author: Colinda Simons, PhD; E-mail address: colinda.simons@maastrichtuniversity.nl; Postal address: P.O. Box 616, 6200 MD Maastricht, The Netherlands; Telephone: +31 (0)43 3882876; Fax: +31 (0)43 3884128

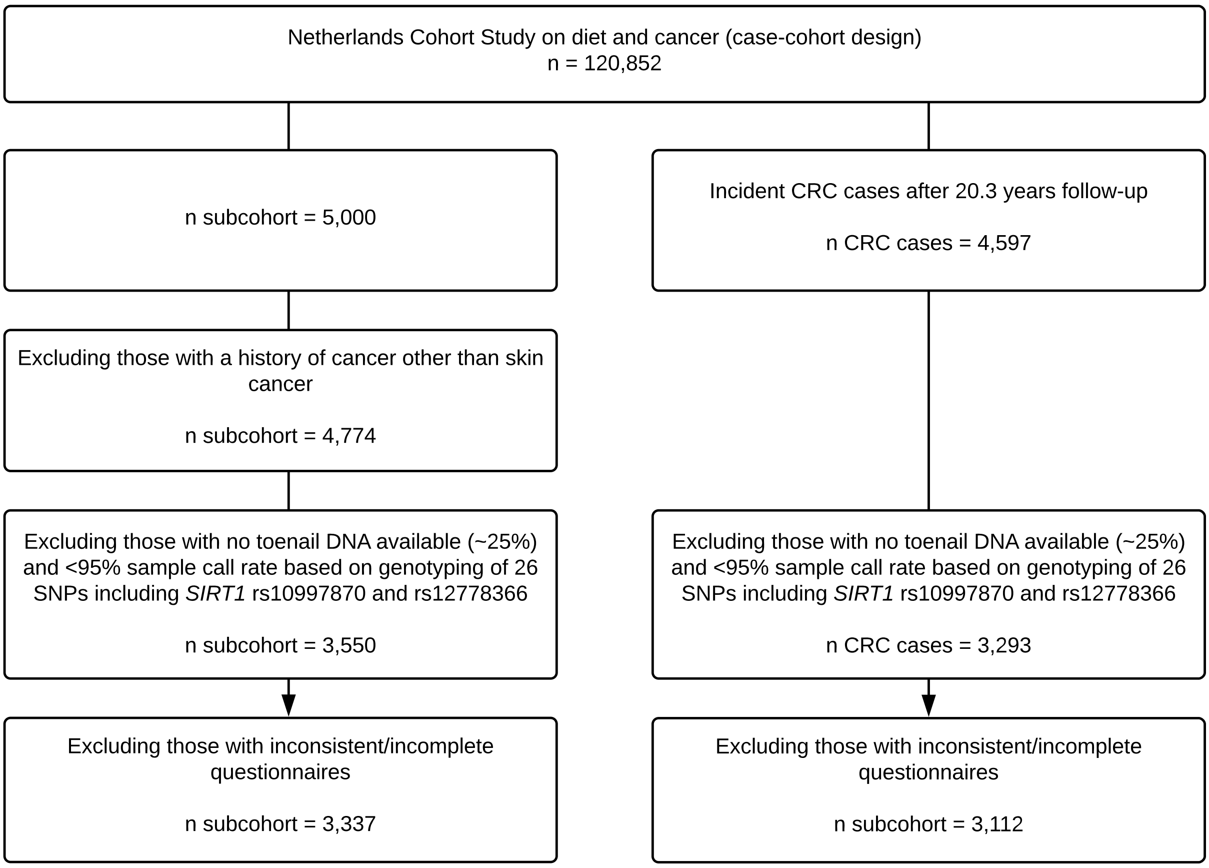

Supplemental Figure 1. Flow chart of subcohort members and colorectal cancer cases with available genotyping information and information on energy balance-related factors.

Supplemental Table 1. Exposures related to energy balance in relation to colon cancer risk in men and women stratified by genotype strata (dominant model) of SIRT1 variants in the Netherlands Cohort Study (20.3 years of follow-up)

		Men										Women									
		BMI										BMI									
		T1 sex-specific (13.5-23.9 kg/m^2)			T2 sex-specific (23.8-25.9 kg/m^2)			T3 sex-specific (25.8-41.3 kg/m^2)				T1 sex-specific (14.5-23.5 kg/m^2)			T2 sex-specific (23.4-26.2 kg/m^2)			T3 sex-specific (26.1-42.2 kg/m^2)			
		N cases/ PT at risk	HR[a]	(95% CI)	N cases/ PT at risk	HR[a]	(95% CI)	N cases/ PT at risk	HR[a]	(95% CI)	*P* for interaction	N cases/ PT at risk	HR[a]	(95% CI)	N cases/ PT at risk	HR[a]	(95% CI)	N cases/ PT at risk	HR[a]	(95% CI)	*P* for interaction
rs10997870	TT	136/3472	1	(ref.)	137/3641	0.85	(0.61,1.18)	184/3454	1.19	(0.86,1.65)		118/3796	1	(ref.)	132/3045	1.29	(0.92,1.81)	99/3140	0.98	(0.69,1.39)	
	TG/GG	206/5466	1	(ref.)	222/5512	1.00	(0.77,1.30)	225/4670	1.26	(0.96,1.64)	0.79	211/5240	1	(ref.)	173/5675	0.77	(0.59,1.00)	179/5170	0.87	(0.66,1.15)	0.04[b]
rs12778366	TT	244/6169	1	(ref.)	262/6782	0.92	(0.72,1.16)	319/6247	1.19	(0.93,1.51)		235/6608	1	(ref.)	234/5989	1.06	(0.83,1.36)	200/5974	0.90	(0.70,1.16)	
	TC/CC	98/2769	1	(ref.)	97/2371	1.04	(0.70,1.55)	90/1877	1.39	(0.92,2.10)	0.77	94/2427	1	(ref.)	71/2731	0.73	(0.49,1.10)	78/2322	0.99	(0.64,1.53)	0.13
		Trouser/skirt size										Trouser/skirt size									
		<median			≥median							<median			≥median						
		N cases/ PT at risk	HR[a]	(95% CI)	N cases/ PT at risk	HR[a]	(95% CI)				*P* for interaction	N cases/ PT at risk	HR[a]	(95% CI)	N cases/ PT at risk	HR[a]	(95% CI)				*P* for interaction
rs10997870	TT	131/3651	1	(ref.)	290/6091	1.06	(0.77,1.48)					157/4593	1	(ref.)	187/5320	1.05	(0.73,1.51)				
	TG/GG	194/5592	1	(ref.)	397/8745	1.29	(1.00,1.66)				0.83	242/6850	1	(ref.)	316/8964	1.03	(0.77,1.37)				0.79
rs12778366	TT	240/6539	1	(ref.)	515/11144	1.12	(0.89,1.41)					291/8290	1	(ref.)	368/10085	1.07	(0.82,1.39)				
	TC/CC	85/2704	1	(ref.)	172/3692	1.39	(0.95,2.06)				0.30	108/3153	1	(ref.)	135/4185	0.94	(0.62,1.44)				0.53
		BMI @ 20 years										BMI @ 20 years									
		T1 sex-specific (11.3-20.8 kg/m^2)			T2 sex-specific (20.7-22.7 kg/m^2)			T3 sex-specific (22.6-33.1 kg.m^2)				T1 sex-specific (11.2-20.3 kg/m^2)			T2 sex-specific (20.2-22.5 kg/m^2)			T3 sex-specific (22.4-46.9 kg/m^2)			
		N cases/ PT at risk	HR[a]	(95% CI)	N cases/ PT at risk	HR[a]	(95% CI)	N cases/ PT at risk	HR[a]	(95% CI)	*P* for interaction	N cases/ PT at risk	HR[a]	(95% CI)	N cases/ PT at risk	HR[a]	(95% CI)	N cases/ PT at risk	HR[a]	(95% CI)	*P* for interaction
rs10997870	TT	147/2883	1	(ref.)	100/2946	0.57	(0.40,0.82)	132/2616	0.83	(0.57,1.19)		99/3006	1	(ref.)	119/3265	1.16	(0.80,1.68)	106/2892	1.25	(0.82,1.90)	
	TG/GG	179/4459	1	(ref.)	182/4142	1.12	(0.84,1.51)	183/4141	1.08	(0.80,1.46)	0.02[b]	169/4993	1	(ref.)	175/4867	1.08	(0.82,1.43)	162/4723	1.09	(0.81,1.48)	0.94
rs12778366	TT	238/5144	1	(ref.)	213/5209	0.88	(0.68,1.14)	233/4952	0.98	(0.75,1.27)		196/5528	1	(ref.)	212/5986	1.04	(0.80,1.35)	204/5474	1.13	(0.84,1.51)	
	TC/CC	88/2197	1	(ref.)	69/1880	0.89	(0.57,1.38)	82/1805	1.03	(0.65,1.63)	0.95	72/2472	1	(ref.)	82/2146	1.40	(0.90,2.19)	64/2127	1.15	(0.72,1.83)	0.55
		Non-occupational physical activity										Non-occupational physical activity									
		≤30 min/day			>30-60 min/day			>60 min/day				≤30 min/day			>30-60 min/day			>60 min/day			

		N cases/ PT at risk	HR[a]	(95% CI)	N cases/ PT at risk	HR[a]	(95% CI)	N cases/ PT at risk	HR[a]	(95% CI)	*P* for interaction	N cases/ PT at risk	HR[a]	(95% CI)	N cases/ PT at risk	HR[a]	(95% CI)	N cases/ PT at risk	HR[a]	(95% CI)	*P* for interaction
rs10997870	TT	78/1540	1	(ref.)	146/3442	0.84	(0.57,1.23)	233/5584	0.82	(0.57,1.18)		88/1752	1	(ref.)	116/3272	0.71	(0.47,1.05)	145/4957	0.60	(0.41,0.88)	
	TG/GG	95/2559	1	(ref.)	191/5145	0.92	(0.67,1.28)	367/7945	1.17	(0.86,1.57)	0.15	140/3843	1	(ref.)	180/5298	0.94	(0.70,1.26)	243/6943	0.95	(0.73,1.25)	0.12
rs12778366	TT	138/2873	1	(ref.)	249/6509	0.75	(0.57,1.00)	438/9816	0.88	(0.67,1.14)		169/3761	1	(ref.)	212/6103	0.79	(0.59,1.04)	288/8707	0.74	(0.57,0.96)	
	TC/CC	35/1226	1	(ref.)	88/2078	1.43	(0.85,2.39)	162/3712	1.47	(0.91,2.36)	0.09	59/1834	1	(ref.)	84/2453	1.10	(0.71,1.71)	100/3193	0.93	(0.60,1.42)	0.45

		Height										Height									
		T1 sex-specific (147-173 cm)			T2 sex-specific (174-179 cm)			T3 sex-specific (180-202 cm)				T1 sex-specific (140-163 cm)			T2 sex-specific (164-168 cm)			T3 sex-specific (169-200 cm)			
		N cases/ PT at risk	HR[a]	(95% CI)	N cases/ PT at risk	HR[a]	(95% CI)	N cases/ PT at risk	HR[a]	(95% CI)	*P* for interaction	N cases/ PT at risk	HR[a]	(95% CI)	N cases/ PT at risk	HR[a]	(95% CI)	N cases/ PT at risk	HR[a]	(95% CI)	*P* for interaction
rs10997870	TT	139/3843	1	(ref.)	158/3411	1.30	(0.95,1.78)	160/3313	1.49	(1.09,2.05)		120/3808	1	(ref.)	116/3354	1.27	(0.89,1.81)	113/2818	1.39	(0.97,2.01)	
	TG/GG	181/5261	1	(ref.)	209/5055	1.28	(0.98,1.67)	263/5332	1.57	(1.21,2.05)	0.86	182/6212	1	(ref.)	203/5468	1.26	(0.97,1.64)	178/4404	1.39	(1.05,1.85)	0.95
rs12778366	TT	247/6730	1	(ref.)	282/6281	1.25	(0.99,1.58)	296/6187	1.39	(1.10,1.76)		218/7193	1	(ref.)	228/6286	1.27	(0.99,1.63)	223/5092	1.54	(1.18,2.00)	
	TC/CC	73/2374	1	(ref.)	85/2185	1.41	(0.93,2.14)	127/2458	1.93	(1.28,2.91)	0.52	84/2827	1	(ref.)	91/2523	1.22	(0.82,1.82)	68/2130	1.03	(0.67,1.59)	0.34

		Residence Hunger Winter										Residence Hunger Winter									
		Non-Western area			Western rural area			Western city				Non-Western area			Western rural area			Western city			
		N cases/ PT at risk	HR[a]	(95% CI)	N cases/ PT at risk	HR[a]	(95% CI)	N cases/ PT at risk	HR[a]	(95% CI)	*P* for interaction	N cases/ PT at risk	HR[a]	(95% CI)	N cases/ PT at risk	HR[a]	(95% CI)	N cases/ PT at risk	HR[a]	(95% CI)	*P* for interaction
rs10997870	TT	247/4973	1	(ref.)	48/1402	0.78	(0.51,1.18)	90/2177	0.84	(0.60,1.19)		175/5204	1	(ref.)	60/1372	1.44	(0.95,2.19)	95/2914	1.02	(0.72,1.44)	
	TG/GG	313/7945	1	(ref.)	91/1901	1.30	(0.94,1.82)	133/3242	1.03	(0.77,1.36)	0.11	279/8610	1	(ref.)	90/2315	1.25	(0.90,1.72)	165/4172	1.25	(0.96,1.62)	0.36
rs12778366	TT	433/9391	1	(ref.)	91/2267	0.98	(0.71,1.33)	153/3968	0.83	(0.64,1.07)		336/9984	1	(ref.)	111/2662	1.29	(0.96,1.74)	187/4940	1.15	(0.90,1.47)	
	TC/CC	127/3527	1	(ref.)	48/1036	1.32	(0.82,2.13)	70/1451	1.27	(0.83,1.93)	0.15	118/3816	1	(ref.)	39/1025	1.29	(0.77,2.14)	73/2145	1.14	(0.78,1.67)	0.99

		Residence World War 2									Residence World War 2						
		Rural area			Urban area				Rural area			Urban area			
		N cases/ PT at risk	HR[a]	(95% CI)	N cases/ PT at risk	HR[a]	(95% CI)	*P* for interaction	N cases/ PT at risk	HR[a]	(95% CI)	N cases/ PT at risk	HR[a]	(95% CI)	*P* for interaction
rs10997870	TT	155/3885	1	(ref.)	189/4116	1.20	(0.88,1.64)		137/3632	1	(ref.)	126/4124	0.79	(0.56,1.10)	
	TG/GG	233/5734	1	(ref.)	242/5679	1.04	(0.82,1.34)	0.69	201/5466	1	(ref.)	240/6157	1.07	(0.83,1.39)	0.14
rs12778366	TT	291/7058	1	(ref.)	313/7304	1.04	(0.83,1.30)		260/6645	1	(ref.)	258/7228	0.90	(0.71,1.15)	

	TC/CC	97/2560	1	(ref.)	118/2491	1.32	(0.90,1.94)	0.40	78/2453	1	(ref.)	108/3052	1.17	(0.79,1.74)	0.33
		Employment status father Economic Depression							Employment status father Economic Depression						
		Employed			Unemployed				Employed			Unemployed			
		N cases/ PT at risk	HR[a]	(95% CI)	N cases/ PT at risk	HR[a]	(95% CI)	*P* for interaction	N cases/ PT at risk	HR[a]	(95% CI)	N cases/ PT at risk	HR[a]	(95% CI)	*P* for interaction
rs10997870	TT	396/9230	1	(ref.)	47/927	1.20	(0.77,1.85)		300/8710	1	(ref.)	33/994	0.98	(0.60,1.61)	
	TG/GG	558/13333	1	(ref.)	75/1644	1.02	(0.72,1.43)	0.52	486/13459	1	(ref.)	56/1662	0.92	(0.64,1.32)	0.85
rs12778366	TT	709/16567	1	(ref.)	89/1842	1.06	(0.78,1.44)		578/15873	1	(ref.)	64/1853	0.94	(0.66,1.33)	
	TC/CC	245/5996	1	(ref.)	33/728	1.13	(0.66,1.92)	0.95	208/6282	1	(ref.)	25/803	0.97	(0.56,1.67)	0.91

Abbreviations: BMI, body mass index; CI, confidence interval; HR, hazard ratio; N, number of; PT, person-time; ref., reference; T1-3, tertile 1-3.

[a] Adjusted for age (years), first-degree family history of colorectal cancer (yes/no), smoking status (never, ex, current), alcohol intake (0, 0.1-29, ≥30 g/d), meat intake (g/d), processed meat intake (g/d), and total energy intake (kcal/d); all models except models for physical activity were additionally adjusted for physical activity (≤30, >30-60, >60 min/day); all models, except models for BMI and physical activity, were additionally adjusted for baseline BMI (kg/m^2).

[b] Remained significant after comparison with the *p*-value adjusted for the Benjamini and Hochberg false discovery rate, setting the false discovery threshold at 0.20.

Supplemental Table 2. Exposures related to energy balance in relation to proximal colon cancer risk in men and women stratified by genotype strata (dominant model) of SIRT1 variants in the Netherlands Cohort Study (20.3 years of follow-up)

		Men										Women									
		BMI										BMI									
		T1 sex-specific (13.5-23.9 kg/m²)			T2 sex-specific (23.8-25.9 kg/m²)			T3 sex-specific (25.8-41.3 kg/m²)				T1 sex-specific (14.5-23.5 kg/m²)			T2 sex-specific (23.4-26.2 kg/m²)			T3 sex-specific (26.1-42.2 kg/m²)			
		N cases/ PT at risk	HR[a]	(95% CI)	N cases/ PT at risk	HR[a]	(95% CI)	N cases/ PT at risk	HR[a]	(95% CI)	*P* for interaction	N cases/ PT at risk	HR[a]	(95% CI)	N cases/ PT at risk	HR[a]	(95% CI)	N cases/ PT at risk	HR[a]	(95% CI)	*P* for interaction
rs10997870	TT	59/3472	1	(ref.)	65/3641	0.92	(0.60,1.42)	85/3454	1.26	(0.82,1.92)		75/3796	1	(ref.)	82/3045	1.25	(0.84,1.85)	56/3140	0.90	(0.59,1.37)	
	TG/GG	94/5466	1	(ref.)	105/5512	1.06	(0.75,1.49)	108/4670	1.38	(0.98,1.93)	0.95	125/5240	1	(ref.)	105/5675	0.79	(0.57,1.09)	97/5170	0.80	(0.57,1.12)	0.17
rs12778366	TT	106/6169	1	(ref.)	123/6782	1.00	(0.73,1.37)	151/6247	1.29	(0.95,1.76)		148/6608	1	(ref.)	146/5989	1.05	(0.79,1.40)	110/5974	0.80	(0.59,1.08)	
	TC/CC	47/2769	1	(ref.)	47/2371	1.02	(0.61,1.71)	42/1877	1.47	(0.86,2.50)	0.88	52/2427	1	(ref.)	41/2731	0.80	(0.48,1.33)	43/2322	1.01	(0.58,1.75)	0.26
		Trouser/skirt size										Trouser/skirt size									
		<median			≥median							<median			≥median						
		N cases/ PT at risk	HR[a]	(95% CI)	N cases/ PT at risk	HR[a]	(95% CI)				*P* for interaction	N cases/ PT at risk	HR[a]	(95% CI)	N cases/ PT at risk	HR[a]	(95% CI)				*P* for interaction
rs10997870	TT	64/3651	1	(ref.)	129/6091	0.95	(0.62,1.46)					97/4593	1	(ref.)	113/5320	1.07	(0.70,1.63)				
	TG/GG	89/5592	1	(ref.)	186/8745	1.29	(0.92,1.80)				0.45	148/6850	1	(ref.)	177/8964	0.87	(0.61,1.22)				0.58
rs12778366	TT	113/6539	1	(ref.)	235/11144	1.06	(0.79,1.44)					182/8290	1	(ref.)	217/10085	1.01	(0.73,1.39)				
	TC/CC	40/2704	1	(ref.)	80/3692	1.35	(0.81,2.25)				0.38	63/3153	1	(ref.)	73/4185	0.78	(0.47,1.29)				0.55
		BMI @ 20 years										BMI @ 20 years									
		T1 sex-specific (11.3-20.8 kg/m²)			T2 sex-specific (20.7-22.7 kg/m²)			T3 sex-specific (22.6-33.1 kg.m²)				T1 sex-specific (11.2-20.3 kg/m²)			T2 sex-specific (20.2-22.5 kg/m²)			T3 sex-specific (22.4-46.9 kg/m²)			
		N cases/ PT at risk	HR[a]	(95% CI)	N cases/ PT at risk	HR[a]	(95% CI)	N cases/ PT at risk	HR[a]	(95% CI)	*P* for interaction	N cases/ PT at risk	HR[a]	(95% CI)	N cases/ PT at risk	HR[a]	(95% CI)	N cases/ PT at risk	HR[a]	(95% CI)	*P* for interaction
rs10997870	TT	73/2883	1	(ref.)	50/2946	0.56	(0.36,0.87)	48/2616	0.59	(0.36,0.96)		63/3006	1	(ref.)	68/3265	1.09	(0.70,1.71)	63/2892	1.21	(0.75,1.98)	
	TG/GG	85/4459	1	(ref.)	89/4142	1.18	(0.81,1.72)	73/4141	0.89	(0.60,1.33)	0.06	97/4993	1	(ref.)	107/4867	1.14	(0.82,1.60)	92/4723	1.07	(0.74,1.54)	0.88
rs12778366	TT	117/5144	1	(ref.)	106/5209	0.87	(0.63,1.20)	82/4952	0.67	(0.48,0.96)		123/5528	1	(ref.)	131/5986	1.04	(0.76,1.41)	114/5474	1.02	(0.72,1.43)	
	TC/CC	41/2197	1	(ref.)	33/1880	0.93	(0.52,1.66)	39/1805	1.06	(0.58,1.92)	0.34	37/2472	1	(ref.)	44/2146	1.53	(0.87,2.69)	41/2127	1.46	(0.81,2.62)	0.58
		Non-occupational physical activity										Non-occupational physical activity									
		≤30 min/day			>30-60 min/day			>60 min/day				≤30 min/day			>30-60 min/day			>60 min/day			

		N cases/ PT at risk	HR[a]	(95% CI)	N cases/ PT at risk	HR[a]	(95% CI)	N cases/ PT at risk	HR[a]	(95% CI)	*P* for interaction	N cases/ PT at risk	HR[a]	(95% CI)	N cases/ PT at risk	HR[a]	(95% CI)	N cases/ PT at risk	HR[a]	(95% CI)	*P* for interaction
rs10997870	TT	40/1540	1	(ref.)	68/3442	0.75	(0.47,1.21)	101/5584	0.68	(0.43,1.07)		47/1752	1	(ref.)	75/3272	0.86	(0.53,1.38)	91/4957	0.71	(0.45,1.11)	
	TG/GG	48/2559	1	(ref.)	94/5145	0.89	(0.59,1.35)	165/7945	1.02	(0.69,1.50)	0.29	80/3843	1	(ref.)	104/5298	0.97	(0.68,1.37)	143/6943	0.99	(0.71,1.37)	0.41
rs12778366	TT	74/2873	1	(ref.)	117/6509	0.66	(0.46,0.94)	189/9816	0.71	(0.51,0.98)		94/3761	1	(ref.)	130/6103	0.86	(0.62,1.20)	180/8707	0.84	(0.62,1.14)	
	TC/CC	14/1226	1	(ref.)	45/2078	1.79	(0.88,3.67)	77/3712	1.71	(0.88,3.32)	0.03[b]	33/1834	1	(ref.)	49/2453	1.18	(0.69,2.02)	54/3193	0.86	(0.50,1.49)	0.63
		Height										Height									
		T1 sex-specific (147-173 cm)			T2 sex-specific (174-179 cm)			T3 sex-specific (180-202 cm)				T1 sex-specific (140-163 cm)			T2 sex-specific (164-168 cm)			T3 sex-specific (169-200 cm)			
		N cases/ PT at risk	HR[a]	(95% CI)	N cases/ PT at risk	HR[a]	(95% CI)	N cases/ PT at risk	HR[a]	(95% CI)	*P* for interaction	N cases/ PT at risk	HR[a]	(95% CI)	N cases/ PT at risk	HR[a]	(95% CI)	N cases/ PT at risk	HR[a]	(95% CI)	*P* for interaction
rs10997870	TT	66/3843	1	(ref.)	70/3411	1.22	(0.82,1.82)	73/3313	1.45	(0.97,2.17)		73/3808	1	(ref.)	71/3354	1.28	(0.84,1.95)	69/2818	1.37	(0.89,2.12)	
	TG/GG	96/5261	1	(ref.)	85/5055	1.02	(0.72,1.44)	126/5332	1.45	(1.04,2.02)	0.67	102/6212	1	(ref.)	131/5468	1.47	(1.08,2.01)	94/4404	1.34	(0.94,1.89)	0.72
rs12778366	TT	125/6730	1	(ref.)	115/6281	1.03	(0.76,1.40)	140/6187	1.35	(1.00,1.80)		126/7193	1	(ref.)	148/6286	1.43	(1.07,1.92)	130/5092	1.54	(1.12,2.10)	
	TC/CC	37/2374	1	(ref.)	40/2185	1.34	(0.78,2.29)	59/2458	1.84	(1.09,3.10)	0.66	49/2827	1	(ref.)	54/2523	1.30	(0.79,2.14)	33/2130	0.88	(0.51,1.52)	0.21
		Residence Hunger Winter										Residence Hunger Winter									
		Non-Western area			Western rural area			Western city				Non-Western area			Western rural area			Western city			
		N cases/ PT at risk	HR[a]	(95% CI)	N cases/ PT at risk	HR[a]	(95% CI)	N cases/ PT at risk	HR[a]	(95% CI)	*P* for interaction	N cases/ PT at risk	HR[a]	(95% CI)	N cases/ PT at risk	HR[a]	(95% CI)	N cases/ PT at risk	HR[a]	(95% CI)	*P* for interaction
rs10997870	TT	120/4973	1	(ref.)	22/1402	0.75	(0.44,1.29)	32/2177	0.63	(0.39,1.01)		103/5204	1	(ref.)	37/1372	1.50	(0.92,2.45)	58/2914	1.02	(0.67,1.55)	
	TG/GG	156/7945	1	(ref.)	47/1901	1.40	(0.92,2.12)	53/3242	0.83	(0.57,1.21)	0.14	147/8610	1	(ref.)	55/2315	1.48	(1.00,2.17)	110/4172	1.61	(1.18,2.18)	0.17
rs12778366	TT	207/9391	1	(ref.)	45/2267	1.03	(0.70,1.53)	60/3968	0.68	(0.49,0.96)		192/9984	1	(ref.)	70/2662	1.43	(1.01,2.03)	118/4940	1.26	(0.94,1.69)	
	TC/CC	69/3527	1	(ref.)	24/1036	1.18	(0.64,2.17)	25/1451	0.82	(0.47,1.46)	0.78	58/3816	1	(ref.)	22/1025	1.53	(0.82,2.89)	50/2145	1.70	(1.06,2.71)	0.60
		Residence World War 2										Residence World War 2									
		Rural area			Urban area							Rural area			Urban area						
		N cases/ PT at risk	HR[a]	(95% CI)	N cases/ PT at risk	HR[a]	(95% CI)				*P* for interaction	N cases/ PT at risk	HR[a]	(95% CI)	N cases/ PT at risk	HR[a]	(95% CI)				*P* for interaction
rs10997870	TT	87/3885	1	(ref.)	77/4116	0.86	(0.58,1.27)					87/3632	1	(ref.)	79/4124	0.76	(0.51,1.13)				
	TG/GG	119/5734	1	(ref.)	104/5679	0.88	(0.64,1.22)				0.69	112/5466	1	(ref.)	150/6157	1.22	(0.90,1.66)				0.07
rs12778366	TT	157/7058	1	(ref.)	132/7304	0.80	(0.61,1.07)					152/6645	1	(ref.)	168/7228	1.01	(0.76,1.33)				

	TC/CC	49/2560	1	(ref.)	49/2491	1.15	(0.69,1.93)	0.36	47/2453	1	(ref.)	61/3052	1.12	(0.68,1.83)	0.83
		Employment status father Economic Depression							Employment status father Economic Depression						
		Employed			Unemployed				Employed			Unemployed			
		N cases/ PT at risk	HR[a]	(95% CI)	N cases/ PT at risk	HR[a]	(95% CI)	*P* for interaction	N cases/ PT at risk	HR[a]	(95% CI)	N cases/ PT at risk	HR[a]	(95% CI)	*P* for interaction
rs10997870	TT	185/9230	1	(ref.)	19/927	1.02	(0.57,1.83)		184/8710	1	(ref.)	21/994	0.99	(0.56,1.76)	
	TG/GG	264/13333	1	(ref.)	31/1644	0.90	(0.57,1.41)	0.66	278/13459	1	(ref.)	33/1662	0.95	(0.61,1.47)	0.81
rs12778366	TT	330/16567	1	(ref.)	38/1842	0.97	(0.65,1.45)		351/15873	1	(ref.)	37/1853	0.88	(0.58,1.33)	
	TC/CC	119/5996	1	(ref.)	12/728	0.91	(0.44,1.89)	0.66	111/6282	1	(ref.)	17/803	1.22	(0.65,2.31)	0.35

Abbreviations: BMI, body mass index; CI, confidence interval; HR, hazard ratio; N, number of; PT, person-time; ref., reference; T1-3, tertile 1-3.

[a] Adjusted for age (years), first-degree family history of colorectal cancer (yes/no), smoking status (never, ex, current), alcohol intake (0, 0.1-29, ≥30 g/d), meat intake (g/d), processed meat intake (g/d), and total energy intake (kcal/d); all models except models for physical activity were additionally adjusted for physical activity (≤30, >30-60, >60 min/day); all models, except models for BMI and physical activity, were additionally adjusted for baseline BMI (kg/m^2).

[b] Remained significant after comparison with the *p*-value adjusted for the Benjamini and Hochberg false discovery rate, setting the false discovery threshold at 0.20.

Supplemental Table 3. Exposures related to energy balance in relation to distal colon cancer risk in men and women stratified by genotype strata (dominant model) of SIRT1 variants in the Netherlands Cohort Study (20.3 years of follow-up)

		Men										Women									
		BMI										BMI									
		T1 sex-specific (13.5-23.9 kg/m^2)			T2 sex-specific (23.8-25.9 kg/m^2)			T3 sex-specific (25.8-41.3 kg/m^2)				T1 sex-specific (14.5-23.5 kg/m^2)			T2 sex-specific (23.4-26.2 kg/m^2)			T3 sex-specific (26.1-42.2 kg/m^2)			
		N cases/ PT at risk	HR[a]	(95% CI)	N cases/ PT at risk	HR[a]	(95% CI)	N cases/ PT at risk	HR[a]	(95% CI)	*P* for interaction	N cases/ PT at risk	HR[a]	(95% CI)	N cases/ PT at risk	HR[a]	(95% CI)	N cases/ PT at risk	HR[a]	(95% CI)	*P* for interaction
rs10997870	TT	73/3472	1	(ref.)	66/3641	0.77	(0.51,1.16)	97/3454	1.17	(0.78,1.75)		41/3796	1	(ref.)	49/3045	1.42	(0.88,2.30)	41/3140	1.11	(0.68,1.81)	
	TG/GG	106/5466	1	(ref.)	107/5512	0.94	(0.68,1.29)	113/4670	1.20	(0.86,1.66)	0.73	78/5240	1	(ref.)	64/5675	0.76	(0.52,1.11)	76/5170	0.99	(0.68,1.45)	0.11
rs12778366	TT	131/6169	1	(ref.)	124/6782	0.80	(0.60,1.08)	164/6247	1.14	(0.85,1.53)		82/6608	1	(ref.)	86/5989	1.13	(0.80,1.60)	86/5974	1.10	(0.78,1.56)	
	TC/CC	48/2769	1	(ref.)	49/2371	1.12	(0.68,1.85)	46/1877	1.36	(0.82,2.26)	0.49	37/2427	1	(ref.)	27/2731	0.65	(0.36,1.15)	31/2322	0.91	(0.51,1.63)	0.21
		Trouser/skirt size										Trouser/skirt size									
		<median			≥median							<median			≥median						
		N cases/ PT at risk	HR[a]	(95% CI)	N cases/ PT at risk	HR[a]	(95% CI)				*P* for interaction	N cases/ PT at risk	HR[a]	(95% CI)	N cases/ PT at risk	HR[a]	(95% CI)				*P* for interaction
rs10997870	TT	63/3651	1	(ref.)	155/6091	1.17	(0.78,1.76)					58/4593	1	(ref.)	71/5320	1.02	(0.62,1.70)				
	TG/GG	101/5592	1	(ref.)	197/8745	1.24	(0.90,1.70)				0.55	86/6850	1	(ref.)	129/8964	1.30	(0.88,1.93)				0.80
rs12778366	TT	120/6539	1	(ref.)	265/11144	1.15	(0.86,1.53)					104/8290	1	(ref.)	145/10085	1.15	(0.79,1.66)				
	TC/CC	44/2704	1	(ref.)	87/3692	1.36	(0.84,2.21)				0.52	40/3153	1	(ref.)	55/4185	1.25	(0.69,2.26)				0.65
		BMI @ 20 years										BMI @ 20 years									
		T1 sex-specific (11.3-20.8 kg/m^2)			T2 sex-specific (20.7-22.7 kg/m^2)			T3 sex-specific (22.6-33.1 kg.m^2)				T1 sex-specific (11.2-20.3 kg/m^2)			T2 sex-specific (20.2-22.5 kg/m^2)			T3 sex-specific (22.4-46.9 kg/m^2)			
		N cases/ PT at risk	HR[a]	(95% CI)	N cases/ PT at risk	HR[a]	(95% CI)	N cases/ PT at risk	HR[a]	(95% CI)	*P* for interaction	N cases/ PT at risk	HR[a]	(95% CI)	N cases/ PT at risk	HR[a]	(95% CI)	N cases/ PT at risk	HR[a]	(95% CI)	*P* for interaction
rs10997870	TT	71/2883	1	(ref.)	49/2946	0.58	(0.37,0.93)	80/2616	1.04	(0.67,1.63)		36/3006	1	(ref.)	47/3265	1.18	(0.71,1.97)	42/2892	1.32	(0.73,2.40)	
	TG/GG	87/4459	1	(ref.)	87/4142	1.12	(0.77,1.62)	106/4141	1.32	(0.92,1.90)	0.12	65/4993	1	(ref.)	63/4867	1.01	(0.68,1.50)	65/4723	1.12	(0.74,1.71)	0.90
rs12778366	TT	114/5144	1	(ref.)	102/5209	0.89	(0.64,1.24)	144/4952	1.28	(0.93,1.77)		71/5528	1	(ref.)	75/5986	1.00	(0.69,1.44)	87/5474	1.28	(0.85,1.93)	
	TC/CC	44/2197	1	(ref.)	34/1880	0.89	(0.52,1.54)	42/1805	1.11	(0.63,1.95)	0.85	30/2472	1	(ref.)	35/2146	1.33	(0.72,2.44)	20/2127	0.83	(0.43,1.61)	0.11
		Non-occupational physical activity										Non-occupational physical activity									
		≤30 min/day			>30-60 min/day			>60 min/day				≤30 min/day			>30-60 min/day			>60 min/day			

		N cases/ PT at risk	HR[a]	(95% CI)	N cases/ PT at risk	HR[a]	(95% CI)	N cases/ PT at risk	HR[a]	(95% CI)	*P* for interaction
rs10997870	TT	36/1540	1	(ref.)	75/3442	0.94	(0.58,1.54)	125/5584	0.97	(0.62,1.54)	
	TG/GG	44/2559	1	(ref.)	88/5145	0.92	(0.60,1.40)	194/7945	1.33	(0.91,1.96)	0.20
rs12778366	TT	59/2873	1	(ref.)	122/6509	0.87	(0.60,1.25)	238/9816	1.11	(0.79,1.56)	
	TC/CC	21/1226	1	(ref.)	41/2078	1.11	(0.59,2.07)	81/3712	1.23	(0.70,2.18)	0.76
		Height									
		T1 sex-specific (147-173 cm)			T2 sex-specific (174-179 cm)			T3 sex-specific (180-202 cm)			
		N cases/ PT at risk	HR[a]	(95% CI)	N cases/ PT at risk	HR[a]	(95% CI)	N cases/ PT at risk	HR[a]	(95% CI)	*P* for interaction
rs10997870	TT	69/3843	1	(ref.)	86/3411	1.42	(0.96,2.10)	81/3313	1.52	(1.03,2.26)	
	TG/GG	82/5261	1	(ref.)	116/5055	1.51	(1.08,2.12)	128/5332	1.66	(1.18,2.33)	0.87
rs12778366	TT	116/6730	1	(ref.)	158/6281	1.45	(1.08,1.94)	145/6187	1.42	(1.06,1.91)	
	TC/CC	35/2374	1	(ref.)	44/2185	1.47	(0.87,2.48)	64/2458	1.91	(1.14,3.20)	0.55
		Residence Hunger Winter									
		Non-Western area			Western rural area			Western city			
		N cases/ PT at risk	HR[a]	(95% CI)	N cases/ PT at risk	HR[a]	(95% CI)	N cases/ PT at risk	HR[a]	(95% CI)	*P* for interaction
rs10997870	TT	126/4973	1	(ref.)	26/1402	0.82	(0.49,1.37)	49/2177	0.89	(0.59,1.35)	
	TG/GG	149/7945	1	(ref.)	42/1901	1.23	(0.81,1.88)	73/3242	1.17	(0.83,1.64)	0.28
rs12778366	TT	220/9391	1	(ref.)	44/2267	0.90	(0.61,1.34)	79/3968	0.83	(0.61,1.13)	
	TC/CC	55/3527	1	(ref.)	24/1036	1.58	(0.88,2.83)	43/1451	1.82	(1.11,2.98)	0.02[b]
		Residence World War 2									
		Rural area			Urban area						
		N cases/ PT at risk	HR[a]	(95% CI)	N cases/ PT at risk	HR[a]	(95% CI)				*P* for interaction
rs10997870	TT	67/3885	1	(ref.)	103/4116	1.54	(1.04,2.29)				
	TG/GG	111/5734	1	(ref.)	125/5679	1.12	(0.82,1.53)				0.29
rs12778366	TT	131/7058	1	(ref.)	164/7304	1.23	(0.93,1.62)				

		N cases/ PT at risk	HR[a]	(95% CI)	N cases/ PT at risk	HR[a]	(95% CI)	N cases/ PT at risk	HR[a]	(95% CI)	*P* for interaction
rs10997870	TT	40/1752	1	(ref.)	41/3272	0.54	(0.31,0.93)	50/4957	0.45	(0.27,0.75)	
	TG/GG	55/3843	1	(ref.)	71/5298	0.92	(0.61,1.37)	92/6943	0.90	(0.62,1.31)	0.07
rs12778366	TT	72/3761	1	(ref.)	81/6103	0.70	(0.48,1.03)	101/8707	0.60	(0.42,0.86)	
	TC/CC	23/1834	1	(ref.)	31/2453	1.00	(0.53,1.86)	41/3193	1.03	(0.57,1.85)	0.30
		Height									
		T1 sex-specific (140-163 cm)			T2 sex-specific (164-168 cm)			T3 sex-specific (169-200 cm)			
		N cases/ PT at risk	HR[a]	(95% CI)	N cases/ PT at risk	HR[a]	(95% CI)	N cases/ PT at risk	HR[a]	(95% CI)	*P* for interaction
rs10997870	TT	46/3808	1	(ref.)	44/3354	1.26	(0.76,2.08)	41/2818	1.37	(0.83,2.27)	
	TG/GG	73/6212	1	(ref.)	69/5468	1.06	(0.73,1.54)	76/4404	1.45	(0.99,2.12)	0.68
rs12778366	TT	89/7193	1	(ref.)	79/6286	1.08	(0.76,1.53)	86/5092	1.48	(1.03,2.12)	
	TC/CC	30/2827	1	(ref.)	34/2523	1.21	(0.69,2.12)	31/2130	1.28	(0.70,2.31)	0.83
		Residence Hunger Winter									
		Non-Western area			Western rural area			Western city			
		N cases/ PT at risk	HR[a]	(95% CI)	N cases/ PT at risk	HR[a]	(95% CI)	N cases/ PT at risk	HR[a]	(95% CI)	*P* for interaction
rs10997870	TT	70/5204	1	(ref.)	21/1372	1.26	(0.70,2.26)	36/2914	1.00	(0.62,1.62)	
	TG/GG	120/8610	1	(ref.)	32/2315	0.98	(0.62,1.55)	53/4172	0.90	(0.63,1.30)	0.87
rs12778366	TT	138/9984	1	(ref.)	38/2662	1.05	(0.69,1.59)	67/4940	1.00	(0.71,1.41)	
	TC/CC	52/3816	1	(ref.)	15/1025	1.06	(0.53,2.14)	22/2145	0.72	(0.41,1.26)	0.79
		Residence World War 2									
		Rural area			Urban area						
		N cases/ PT at risk	HR[a]	(95% CI)	N cases/ PT at risk	HR[a]	(95% CI)				*P* for interaction
rs10997870	TT	49/3632	1	(ref.)	44/4124	0.78	(0.47,1.27)				
	TG/GG	85/5466	1	(ref.)	83/6157	0.86	(0.60,1.23)				0.69
rs12778366	TT	105/6645	1	(ref.)	84/7228	0.72	(0.51,1.01)				

	TC/CC	47/2560	1	(ref.)	64/2491	1.39	(0.87,2.22)	0.66	29/2453	1	(ref.)	43/3052	1.21	(0.70,2.10)	0.12

		Employment status father Economic Depression							Employment status father Economic Depression						
		Employed			Unemployed				Employed			Unemployed			
		N cases/ PT at risk	HR[a]	(95% CI)	N cases/ PT at risk	HR[a]	(95% CI)	*P* for interaction	N cases/ PT at risk	HR[a]	(95% CI)	N cases/ PT at risk	HR[a]	(95% CI)	*P* for interaction
rs10997870	TT	201/9230	1	(ref.)	27/927	1.36	(0.82,2.28)		113/8710	1	(ref.)	11/994	0.89	(0.43,1.84)	
	TG/GG	278/13333	1	(ref.)	41/1644	1.11	(0.74,1.68)	0.52	192/13459	1	(ref.)	21/1662	0.87	(0.52,1.45)	0.94
rs12778366	TT	357/16567	1	(ref.)	49/1842	1.17	(0.81,1.69)		218/15873	1	(ref.)	26/1853	1.04	(0.65,1.66)	
	TC/CC	122/5996	1	(ref.)	19/728	1.21	(0.64,2.29)	0.99	87/6282	1	(ref.)	6/803	0.57	(0.23,1.40)	0.22

Abbreviations: BMI, body mass index; CI, confidence interval; HR, hazard ratio; N, number of; PT, person-time; ref., reference; T1-3, tertile 1-3.

[a] Adjusted for age (years), first-degree family history of colorectal cancer (yes/no), smoking status (never, ex, current), alcohol intake (0, 0.1-29, ≥30 g/d), meat intake (g/d), processed meat intake (g/d), and total energy intake (kcal/d); all models except models for physical activity were additionally adjusted for physical activity (≤30, >30-60, >60 min/day); all models, except models for BMI and physical activity, were additionally adjusted for baseline BMI (kg/m^2).

[b] Remained significant after comparison with the *p*-value adjusted for the Benjamini and Hochberg false discovery rate, setting the false discovery threshold at 0.20.

Supplemental Table 4. Exposures related to energy balance in relation to rectal cancer risk in men and women stratified by genotype strata (dominant model) of SIRT1 variants in the Netherlands Cohort Study (20.3 years of follow-up)

Men – BMI

		T1 sex-specific (13.5-23.9 kg/m^2)			T2 sex-specific (23.8-25.9 kg/m^2)			T3 sex-specific (25.8-41.3 kg/m^2)			
		N cases/ PT at risk	HR[a]	(95% CI)	N cases/ PT at risk	HR[a]	(95% CI)	N cases/ PT at risk	HR[a]	(95% CI)	*P* for interaction
rs10997870	TT	68/3472	1	(ref.)	56/3641	0.81	(0.53,1.23)	67/3454	1.03	(0.67,1.58)	
	TG/GG	65/5466	1	(ref.)	98/5512	1.41	(0.98,2.02)	87/4670	1.50	(1.03,2.19)	0.08
rs12778366	TT	102/6169	1	(ref.)	118/6782	1.04	(0.76,1.42)	119/6247	1.13	(0.82,1.55)	
	TC/CC	31/2769	1	(ref.)	36/2371	1.25	(0.70,2.21)	35/1877	1.65	(0.90,3.03)	0.57

Women – BMI

		T1 sex-specific (14.5-23.5 kg/m^2)			T2 sex-specific (23.4-26.2 kg/m^2)			T3 sex-specific (26.1-42.2 kg/m^2)			
		N cases/ PT at risk	HR[a]	(95% CI)	N cases/ PT at risk	HR[a]	(95% CI)	N cases/ PT at risk	HR[a]	(95% CI)	*P* for interaction
rs10997870	TT	33/3796	1	(ref.)	29/3045	1.02	(0.58,1.80)	28/3140	0.97	(0.55,1.70)	
	TG/GG	46/5240	1	(ref.)	36/5675	0.68	(0.42,1.10)	44/5170	0.91	(0.56,1.47)	0.52
rs12778366	TT	58/6608	1	(ref.)	51/5989	0.91	(0.60,1.38)	54/5974	0.95	(0.63,1.43)	
	TC/CC	21/2427	1	(ref.)	14/2731	0.62	(0.29,1.32)	18/2322	1.02	(0.47,2.23)	0.51

Men – Trouser/skirt size

		<median			≥median			
		N cases/ PT at risk	HR[a]	(95% CI)	N cases/ PT at risk	HR[a]	(95% CI)	*P* for interaction
rs10997870	TT	73/3651	1	(ref.)	102/6091	0.75	(0.50,1.12)	
	TG/GG	76/5592	1	(ref.)	152/8745	1.26	(0.89,1.78)	0.06
rs12778366	TT	114/6539	1	(ref.)	201/11144	1.03	(0.76,1.38)	
	TC/CC	35/2704	1	(ref.)	53/3692	1.01	(0.58,1.73)	0.73

Women – Trouser/skirt size

		<median			≥median			
		N cases/ PT at risk	HR[a]	(95% CI)	N cases/ PT at risk	HR[a]	(95% CI)	*P* for interaction
rs10997870	TT	39/4593	1	(ref.)	48/5320	1.21	(0.70,2.09)	
	TG/GG	54/6850	1	(ref.)	71/8964	0.82	(0.49,1.37)	0.80
rs12778366	TT	70/8290	1	(ref.)	90/10085	0.98	(0.64,1.50)	
	TC/CC	23/3153	1	(ref.)	29/4185	0.92	(0.42,2.00)	0.75

Men – BMI @ 20 years

		T1 sex-specific (11.3-20.8 kg/m^2)			T2 sex-specific (20.7-22.7 kg/m^2)			T3 sex-specific (22.6-33.1 kg.m^2)			
		N cases/ PT at risk	HR[a]	(95% CI)	N cases/ PT at risk	HR[a]	(95% CI)	N cases/ PT at risk	HR[a]	(95% CI)	*P* for interaction
rs10997870	TT	58/2883	1	(ref.)	49/2946	0.82	(0.51,1.32)	52/2616	0.96	(0.59,1.58)	
	TG/GG	64/4459	1	(ref.)	69/4142	1.18	(0.79,1.76)	67/4141	1.03	(0.69,1.56)	0.40
rs12778366	TT	95/5144	1	(ref.)	92/5209	0.99	(0.70,1.40)	93/4952	1.05	(0.73,1.49)	
	TC/CC	27/2197	1	(ref.)	26/1880	1.05	(0.57,1.95)	26/1805	0.88	(0.46,1.66)	0.93

Women – BMI @ 20 years

		T1 sex-specific (11.2-20.3 kg/m^2)			T2 sex-specific (20.2-22.5 kg/m^2)			T3 sex-specific (22.4-46.9 kg/m^2)			
		N cases/ PT at risk	HR[a]	(95% CI)	N cases/ PT at risk	HR[a]	(95% CI)	N cases/ PT at risk	HR[a]	(95% CI)	*P* for interaction
rs10997870	TT	28/3006	1	(ref.)	31/3265	1.02	(0.56,1.86)	27/2892	1.10	(0.56,2.16)	
	TG/GG	44/4993	1	(ref.)	31/4867	0.76	(0.45,1.27)	37/4723	0.84	(0.48,1.46)	0.77
rs12778366	TT	52/5528	1	(ref.)	52/5986	0.93	(0.60,1.45)	46/5474	0.85	(0.50,1.44)	
	TC/CC	20/2472	1	(ref.)	10/2146	0.67	(0.27,1.64)	18/2127	1.08	(0.51,2.30)	0.41

Men – Non-occupational physical activity

≤30 min/day	>30-60 min/day	>60 min/day

Women – Non-occupational physical activity

≤30 min/day	>30-60 min/day	>60 min/day

		N cases/ PT at risk	HR[a]	(95% CI)	N cases/ PT at risk	HR[a]	(95% CI)	N cases/ PT at risk	HR[a]	(95% CI)	*P* for interaction	N cases/ PT at risk	HR[a]	(95% CI)	N cases/ PT at risk	HR[a]	(95% CI)	N cases/ PT at risk	HR[a]	(95% CI)	*P* for interaction
rs10997870	TT	32/1540	1	(ref.)	53/3442	0.73	(0.44,1.22)	106/5584	0.87	(0.54,1.40)		26/1752	1	(ref.)	25/3272	0.53	(0.29,0.99)	39/4957	0.51	(0.29,0.90)	
	TG/GG	35/2559	1	(ref.)	72/5145	1.01	(0.64,1.60)	143/7945	1.31	(0.86,1.99)	0.46	41/3843	1	(ref.)	35/5298	0.64	(0.39,1.05)	50/6943	0.71	(0.45,1.13)	0.76
rs12778366	TT	53/2873	1	(ref.)	101/6509	0.83	(0.56,1.22)	185/9816	1.00	(0.70,1.42)		52/3761	1	(ref.)	45/6103	0.55	(0.35,0.85)	66/8707	0.55	(0.37,0.83)	
	TC/CC	14/1226	1	(ref.)	24/2078	1.05	(0.49,2.23)	64/3712	1.51	(0.78,2.91)	0.51	15/1834	1	(ref.)	15/2453	0.78	(0.36,1.71)	23/3193	0.82	(0.38,1.74)	0.61
		Height										Height									
		T1 sex-specific (147-173 cm)			T2 sex-specific (174-179 cm)			T3 sex-specific (180-202 cm)				T1 sex-specific (140-163 cm)			T2 sex-specific (164-168 cm)			T3 sex-specific (169-200 cm)			
		N cases/ PT at risk	HR[a]	(95% CI)	N cases/ PT at risk	HR[a]	(95% CI)	N cases/ PT at risk	HR[a]	(95% CI)	*P* for interaction	N cases/ PT at risk	HR[a]	(95% CI)	N cases/ PT at risk	HR[a]	(95% CI)	N cases/ PT at risk	HR[a]	(95% CI)	*P* for interaction
rs10997870	TT	79/3843	1	(ref.)	59/3411	0.83	(0.56,1.23)	53/3313	0.73	(0.48,1.11)		26/3808	1	(ref.)	32/3354	1.54	(0.86,2.77)	32/2818	1.81	(0.98,3.35)	
	TG/GG	80/5261	1	(ref.)	88/5055	1.21	(0.84,1.72)	82/5332	1.08	(0.75,1.55)	0.38	36/6212	1	(ref.)	51/5468	1.67	(1.04,2.69)	39/4404	1.69	(1.02,2.80)	0.84
rs12778366	TT	123/6730	1	(ref.)	109/6281	0.95	(0.70,1.29)	107/6187	0.93	(0.69,1.26)		49/7193	1	(ref.)	62/6286	1.50	(0.99,2.29)	52/5092	1.63	(1.04,2.55)	
	TC/CC	36/2374	1	(ref.)	38/2185	1.23	(0.71,2.13)	28/2458	0.83	(0.46,1.49)	0.45	13/2827	1	(ref.)	21/2523	1.98	(0.91,4.30)	19/2130	1.99	(0.92,4.32)	0.85
		Residence Hunger Winter										Residence Hunger Winter									
		Non-Western area			Western rural area			Western city				Non-Western area			Western rural area			Western city			
		N cases/ PT at risk	HR[a]	(95% CI)	N cases/ PT at risk	HR[a]	(95% CI)	N cases/ PT at risk	HR[a]	(95% CI)	*P* for interaction	N cases/ PT at risk	HR[a]	(95% CI)	N cases/ PT at risk	HR[a]	(95% CI)	N cases/ PT at risk	HR[a]	(95% CI)	*P* for interaction
rs10997870	TT	107/4973	1	(ref.)	20/1402	0.69	(0.39,1.19)	27/2177	0.57	(0.35,0.94)		47/5204	1	(ref.)	12/1372	1.06	(0.51,2.24)	25/2914	0.87	(0.49,1.53)	
	TG/GG	145/7945	1	(ref.)	35/1901	1.11	(0.71,1.73)	47/3242	0.79	(0.54,1.15)	0.36	70/8610	1	(ref.)	19/2315	1.10	(0.63,1.92)	31/4172	0.96	(0.60,1.53)	0.98
rs12778366	TT	198/9391	1	(ref.)	38/2267	0.83	(0.55,1.25)	49/3968	0.58	(0.41,0.83)		88/9984	1	(ref.)	23/2662	1.01	(0.60,1.70)	42/4940	0.98	(0.65,1.48)	
	TC/CC	54/3527	1	(ref.)	17/1036	1.17	(0.60,2.29)	25/1451	1.11	(0.64,1.93)	0.14	29/3816	1	(ref.)	8/1025	1.17	(0.49,2.80)	14/2145	0.88	(0.45,1.75)	0.97
		Residence World War 2										Residence World War 2									
		Rural area			Urban area							Rural area			Urban area						
		N cases/ PT at risk	HR[a]	(95% CI)	N cases/ PT at risk	HR[a]	(95% CI)				*P* for interaction	N cases/ PT at risk	HR[a]	(95% CI)	N cases/ PT at risk	HR[a]	(95% CI)				*P* for interaction
rs10997870	TT	74/3885	1	(ref.)	77/4116	1.02	(0.69,1.49)					37/3632	1	(ref.)	35/4124	0.87	(0.50,1.52)				
	TG/GG	115/5734	1	(ref.)	90/5679	0.79	(0.57,1.09)				0.37	45/5466	1	(ref.)	43/6157	0.81	(0.51,1.29)				0.98
rs12778366	TT	141/7058	1	(ref.)	127/7304	0.87	(0.65,1.15)					63/6645	1	(ref.)	60/7228	0.90	(0.60,1.34)				

	TC/CC	48/2560	1	(ref.)	40/2491	0.88	(0.52,1.47)	0.95	19/2453	1	(ref.)	18/3052	0.67	(0.32,1.38)	0.70
		Employment status father Economic Depression							Employment status father Economic Depression						
		Employed			Unemployed				Employed			Unemployed			
		N cases/ PT at risk	HR[a]	(95% CI)	N cases/ PT at risk	HR[a]	(95% CI)	*P* for interaction	N cases/ PT at risk	HR[a]	(95% CI)	N cases/ PT at risk	HR[a]	(95% CI)	*P* for interaction
rs10997870	TT	156/9230	1	(ref.)	24/927	1.60	(0.95,2.71)		77/8710	1	(ref.)	9/994	0.94	(0.43,2.08)	
	TG/GG	223/13333	1	(ref.)	20/1644	0.70	(0.41,1.17)	0.03[b]	106/13459	1	(ref.)	11/1662	0.77	(0.39,1.53)	0.70
rs12778366	TT	286/16567	1	(ref.)	36/1842	1.11	(0.74,1.67)		138/15873	1	(ref.)	15/1853	0.87	(0.49,1.57)	
	TC/CC	93/5996	1	(ref.)	8/728	0.74	(0.32,1.72)	0.32	45/6282	1	(ref.)	5/803	0.81	(0.30,2.22)	0.96

Abbreviations: BMI, body mass index; CI, confidence interval; HR, hazard ratio; N, number of; PT, person-time; ref., reference; T1-3, tertile 1-3.

[a] Adjusted for age (years), first-degree family history of colorectal cancer (yes/no), smoking status (never, ex, current), alcohol intake (0, 0.1-29, ≥30 g/d), meat intake (g/d), processed meat intake (g/d), and total energy intake (kcal/d); all models except models for physical activity were additionally adjusted for physical activity (≤30, >30-60, >60 min/day); all models, except models for BMI and physical activity, were additionally adjusted for baseline BMI (kg/m^2).

[b] Remained significant after comparison with the *p*-value adjusted for the Benjamini and Hochberg false discovery rate, setting the false discovery threshold at 0.20.
